# Supplementary material for: Topological phonon transport in an optomechanical system
Source: Nat Commun. 2022 Jun 17;13:3476. doi: 10.1038/s41467-022-30941-0 (PMC9205990; doi:10.1038/s41467-022-30941-0)
Supplement: Supplementary file 1 — Supplementary Info [file 41467_2022_30941_MOESM1_ESM.pdf]

Supplementary Information: Topological phonon transport in an optomechanical system

Hengjiang Ren,[1,2,*] Tirth Shah,[3,4,†] Hannes Pfeifer,[3,‡] Christian Brendel,[3] Vittorio Peano,[3] Florian Marquardt,[3,4] and Oskar Painter[1,2,5]

[1]*Thomas J. Watson, Sr., Laboratory of Applied Physics and Kavli Nanoscience Institute, California Institute of Technology, Pasadena, CA 91125, USA*
[2]*Institute for Quantum Information and Matter, California Institute of Technology, Pasadena, CA 91125, USA*
[3]*Max Planck Institute for the Science of Light, Staudtstr. 2, 91058 Erlangen, Germany*
[4]*Department of Physics, Friedrich-Alexander Universität Erlangen-Nürnberg, Staudtstr. 7, 91058 Erlangen, Germany*
[5]*AWS Center for Quantum Computing, Pasadena, CA 91125, USA.*[§]

(Dated: June 13, 2022)

* These authors contributed equally to this work.; Present Address: Institute of High Performance Computing, Agency for Science, Technology and Research (A*STAR), Singapore 138632, and Anyon Computing Inc, Dover, DE 19901, USA
† These authors contributed equally to this work.
‡ Present Address: Institut für Angewandte Physik, Universität Bonn, Wegelerstr. 8, 53115 Bonn, Germany
§ opainter@caltech.edu

Supplementary Note 1. FINITE ELEMENT SIMULATIONS

In Fig.1 of Main Text, we show the phononic band structures and the photonic crystal cavity modes. The mechanical normal modes are obtained by numerically solving the eigenvalue equation,

$$\mathrm{div}\left[\mathbf{C}:\left[\mathrm{grad}\,\mathbf{Q}_n(\mathbf{r})+(\mathrm{grad}\,\mathbf{Q}_n(\mathbf{r}))^T\right]\right]=-2\Omega_n^2\rho(\mathbf{r})\mathbf{Q}_n(\mathbf{r}). \tag{1}$$

Here and throughout the Supplemental Information, $\mathbf{Q}_n(\mathbf{r})$ ($\mathbf{Q}_{\mathbf{k},n}(\mathbf{r})$) denotes the three-dimensional mechanical displacement $\mathbf{Q_n}(\mathbf{r},\mathbf{t})=\mathrm{Re}\left[\mathbf{Q_n}(\mathbf{r})\cdot e^{-i\Omega_n t}\right]$ for a normal mode (Bloch wave) with eigenfrequency Ω_n. Moreover, $\mathbf{C}$ is the elasticity tensor, ρ the mass density, and : is a symbol for the tensor product, where $[\mathbf{C}:\mathrm{grad}\,\boldsymbol{\psi}]_{ij}=C_{ijkl}\partial_l\psi_k$.

Optically, our structure is described by the Maxwell's equations, which in the absence of a source, takes the form of the following eigenvalue equation

$$c^2\mathrm{curl}\left[\frac{1}{\varepsilon(\mathbf{r})}\mathrm{curl}\,\mathbf{H}_n(\mathbf{r})\right]=\omega_n^2\mathbf{H}_n(\mathbf{r}). \tag{2}$$

where $\mathbf{H}_n(\mathbf{r})$ denotes the magnetic field $\mathbf{H_n}(\mathbf{r},\mathbf{t})=\mathrm{Re}\left[\mathbf{H_n}(\mathbf{r})\cdot e^{-i\omega_n t}\right]$ for the photonic crystal cavity mode with eigenfrequency ω_n. c is the speed of light in vacuum, and $\varepsilon(\mathbf{r})$ is the relative permittivity of the medium. Both of these equations are solved with the finite-element method (FEM) solver [1].

Supplementary Note 2. DEVICE FABRICATION AND MEASUREMENT SETUP

The devices were fabricated on silicon-on-insulator (SOI) wafers from SEH, with a device layer Si thickness 220 nm, buried oxide (BOX) layer 3 μm, handle Si thickness 500 μm, crystal orientation $\langle 1,0,0\rangle$, resistivity $\rho>3000\ \Omega\cdot$cm, diced into die of 5×10 mm. Silicon-on-insulator is a natural choice of substrate as it allows the ease of fabrication of suspended nanoscale structures within the thin membrane of the silicon device layer by simply removing or undercutting the buried oxide layer, and the standard fabrication processes for SOI are very mature in both industrial and academic applications. In its simplest form this process involves only a single layer of lithography to fabricate suspended silicon structures. An overview of the fabrication process for the devices is:

1. Pre-cleaning of the chip. This step prepares the substrate chip for application of resist. Chips will typically retain some protective coating after the wafer-dicing process, either in the form of an adhesive film or a layer of photoresist. In either case, a solvent rinse in acetone (ACE) followed by isopropanol (IPA) is usually sufficient to obtain a clean chip surface.

2. Spinning and baking of electron-beam (e-beam) resist, spin speed 8000 rpm, ramp speed 2500rpm/s, spin time 60s. We typically use a ZEON ZEP-520A e-beam resist for its high resolution and high selectivity against common plasma etch chemistries. The resist is hardened by baking using a hot-plate, 180° C, 2 minutes.

3. Electron-beam (e-beam) lithography exposure. The device pattern is defined in the resist using e-beam lithography, including proximity effect corrections to refine the dose exposing the resist. The electron beam dose is 180μ C/cm, beam voltage is 100keV, beam current is 140pA, resolution/beam step size is $1-2.5$ nm.

4. Resist development. The chip is submerged in a developing solvent to relieve the patterns, in the case of ZEP-520A the developer used here is ZED-N50, 2 minutes 30 seconds. Followed by MIBK rinse, 30 seconds.

5. Plasma etching. Inductively-coupled plasma reactive-ion etching (ICP-RIE) is used to transfer the device pattern from the resist into the silicon layer. Etching parameters are listed in Supplementary Table I.

6. Resist stripping. A chemical cleaning is performed to remove the resist layer, typically by submerging the chip in a piranha solution (3:1 sulfuric acid to hydrogen peroxide), in which a highly exothermic reaction chemically burns organics such as resist from the chip and mechanically scrubs debris.

7. Oxide undercutting or device layer *release*. To remove the buried oxide in the vicinity of the OMC device, hydrofluoric acid (HF) is used as an etchant. This is typically performed using anhydrous vapor-HF.

Note that the mechanical frequencies are changed in the tree geometries compared to triangle geometries, because parameters of snowflake structures (a_m, r and w) in the tree geometries have been scaled by an overall factor of 1.01 with respect to the triangle samples (the photonic crystal properties were kept identical).

Supplementary Table 1. ICP-RIE optimized etch recipe parameters.

Parameter	Substrate SOI layer
	Device-layer Si
C_4F_8 flow (sccm)	72
SF_6 flow (sccm)	30
O_2 flow (sccm)	0
RF power (W)	15.5
ICP power (W)	600
D.C. bias (V)	78
Chamber pressure (mTorr)	15
Helium pressure (Torr)	10
Helium flow (sccm)	5.0-6.0
Table temperature (°C)	15
Etch rate (nm/min)	45

In the optical measurement setup, as a light source we use a fiber-coupled, tunable, near-infrared laser, (New Focus Velocity 6728-P-D tunable laser) spanning 1520-1570 nm tuning range, and 30 mW of output power at 1550 nm, which has its intensity controlled by a variable optical attenuator (VOA). The Spectrum Analyzer we used in the setup is Rohde-Schwarz ZNB (100 kHz to 20 GHz), with Spectrum Analyzer mode. Note that the optical power after the polarization controller, before the dimpled fiber taper, is 240μW. The optical power after the dimpled fiber taper is 60.3μW. We assume the efficiencies for both sides of the dimpled fiber taper are the same, and the optical power launched to the cavity is 120.6μW.

The dimpled fiber taper probe is made from a standard straight fiber taper that is pressed against a mold and heated. We form "straight" fiber tapers by simultaneously heating and pulling standard telecommunication fiber (specifically SMF-28e). By slowly thinning the fiber, the fundamental core-guided fiber mode is adiabatically converted to the fundamental taper mode with evanescent tails that extend significantly into the surrounding medium. After mounting the taper in a U-bracket, the narrowest part of the taper is pressed against a silica mold with the desired radius of curvature; a bare optical fiber with a radius of approximately 62 m is used as the mold. The taper and mold are heated with a hydrogen torch and allowed to cool. After detaching the fiber from the mold, the taper retains an impression of the mold, which forms a global minimum with respect the rest of the taper. The dimpling process introduces negligible additional loss, and the total loss of the dimpled taper is typically less than 0.5 dB relative to the un-pulled optical fiber. Using a specially designed U-mount with a set screw to control the tensioning, varying the taper's tension changes the radius of curvature of the dimple. Under high tension, the dimple becomes very shallow but never completely straightens. After dimpling, the probe is mounted onto a three-axis 50-nm-encoded stage and is fusion-spliced into a fiber-optic setup. At tension used in this experiment, shows only 20 m (full width at half max) of the taper at the bottom of the dimple is close enough to interact with the sample. The probe radius is fitted to be 228 m at this tension. These radii most likely differ from the mold radius ($\sim 62\mu$m) because of tensioning and how the fiber detaches from the mold after heating.

Supplementary Note 3. OPTICAL CAVITY DESIGN AND CHARACTERIZATION

The device in this work is designed around the silicon-on-insulator (SOI) materials platform, which naturally provides a thin Si device layer with typical widths of a few hundred nanometers. In our multiscale optomechanical crystal design, photonic crystal optical cavities are embedded inside the triangular membranes forming the larger scale snowflake phononic crystal. The role of the optical cavities is to amplify the radiation pressure force of the laser light. The radiation pressure force is given by $\hbar G|a|^2$, where G is the optomechanical frequency shift per displacement and $|a|^2 = n_\mathrm{c}$ is the number of intra-cavity photons.

Our optical cavity has been engineered starting from an existing design [2]. A cavity based on this design has displayed the highest observed optical quality factor reported in the literature ($Q \sim 10^7$). Here, we have modified the original design to achieve a stronger coupling of the cavity resonance to the Dirac cone mechanical normal modes while maintaining the high optical quality factor. The underlying basic design is a triangular lattice of cylindrical holes. An effective means of forming resonant cavities in such 2D slab photonic crystal structures is to weakly modulate the properties of a line-defect waveguide (W1 waveguide) [3, 4]. Leaky optical resonances are localized inside the slab and yet have wave vector components which radiate energy into the surrounding cladding, which is a major source of

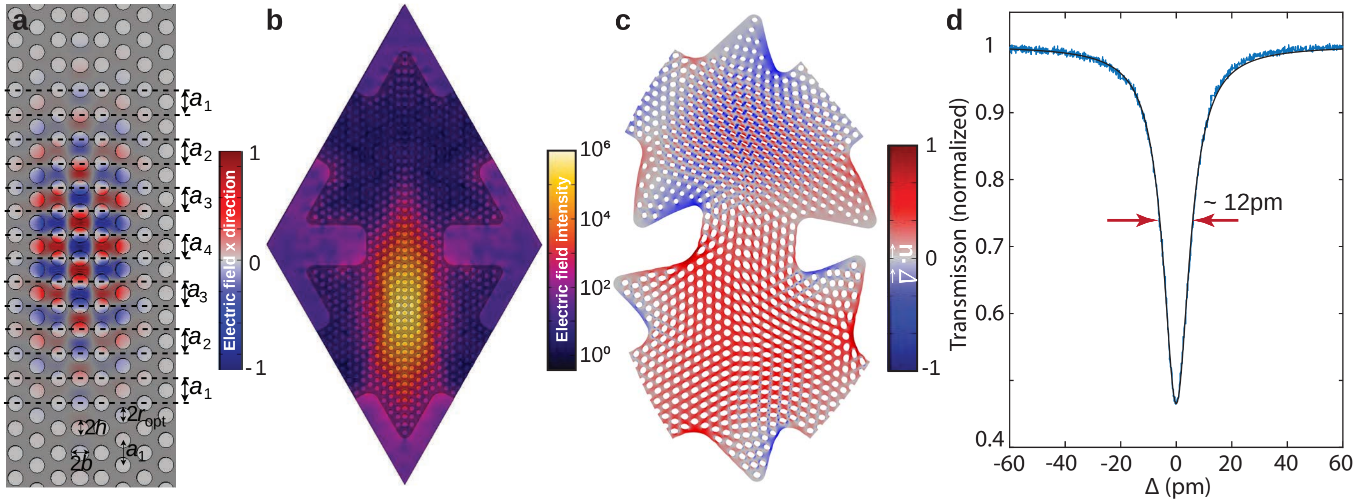

Supplementary Figure 1. **Optical cavity design and characterization.** **a**, Design of optical cavity, local lattice constant is increased smoothly from $a_1 = 450nm$ to $a_4 = 470nm$. **b**, Optimum position of optical cavity in order to simultaneously achieve a large g_0 and optical quality factor. **c**, Snapshots of the mechanical deformation at the degeneracy breaking point for upper band. The colours represent the change of volume ($\boldsymbol{\nabla} \cdot \mathbf{u}$), with red (blue) corresponding to expansion (compression). **d**, Optical spectrum of a optical cavity measured using a swept laser scan.

optical loss in real fabricated structures, causing light scattering out of the plane of the slab. A line-defect waveguide in a triangular lattice of cylindrical holes can be designed to have no leaky mode bands crossing the localized cavity mode frequency. In order to form a localized cavity resonance, the local lattice constant is increased smoothly from a nominal value of $a_1 = 450$ nm to a value of $a_4 = 470$ nm in the center of the cavity (See Supplementary Figure 1a), such that a localized resonance is created from shifting the higher frequency waveguide band into the bandgap.

The Dirac cone mechanical modes are in-plane modes with a breathing motion about the centre of the triangle. The change of volume ($\nabla \cdot \overrightarrow{u}$) of such a mechanical breathing mode, simulated using FEM calculations, is shown in Supplementary Figure 1c. The mechanical breathing modes have naturally large optomechanical couplings, since breathing modes can efficiently interact with the optical cavity by moving the dielectric boundaries near the optical cavity as well as produce strain which overlaps with the electrical field of the optical resonance. In order to boost both the "moving-boundary" and the "photo-elastic" type of optomechanical coupling even further, we have added elliptical holes in the center of the W1 waveguide defect. Moreover, we have optimized the position of the center of the optical cavity along the y-axis in order to increase the optical quality factor (Q_c) within the limited triangular membrane region. This modification contributes as well to an increase of the optomechanical coupling.

The optical properties of the resonances of the optical cavities are determined by scanning the tunable laser across the $\lambda = 1520 - 1570$ nm wavelength band, and measuring the transmitted optical power on a photodetector (PD1, c.f. Main Text Fig.2a). From the normalized transmission spectrum, the wavelength of the optical resonance, the total optical cavity decay rate, and the external coupling rate to the fiber taper waveguide for a device being tested can be determined. An example of the transmission spectrum is shown in Supplementary Figure 1d, where the optical cavity decay rate and external coupling rate are fitted to be $\kappa/2\pi \approx 1.5$ GHz and $\kappa_\mathrm{e}/2\pi \approx 0.47$ GHz, respectively, corresponding to a loaded (extrinsic) optical Q_c factor of $\approx 129k$ ($Q_\mathrm{c,e}$ of $\approx 410k$).

Finally, the unit-cell single-photon optomechanical coupling strengths between the localized optical resonance and the two gapped Dirac cone modes in the unit cell geometry are calculated to be $g_0/2\pi = 5.5$kHz (lower band) and 33.7kHz (upper band).

Supplementary Note 4. THEORETICAL DESCRIPTION OF THE EDGE STATES USING THE DIRAC EQUATION

Here, we derive the Dirac Hamiltonian of the Main Text and solve for its eigenstates and eigenvalues.

Derivation of the massless Dirac Hamiltonian

We first consider the special case in which Dirac cones are gapless. For our discussion only the in-plane symmetries are important. The general framework is a system with Wallpaper group cmm (point group $\mathcal{C}_{2\nu}$) with a pair of Dirac cones on the k_x-axis. Since the two valleys are mapped one onto the other by the time-reversal symmetry it is entirely sufficient to discuss the dynamics for just one valley. Most of the discussion will be general but, when concreteness requires it, we focus on the valley whose degeneracy point lies on the positive k_x-axis in the first Brillouin zone (BZ).

For the cmm scenario, the point group includes the mirror symmetry M_y (with the mirror plane zx, flipping $y \mapsto -y$). Since M_y does not change the quasi-momentum on the k_x axis, the Bloch waves can be chosen to be its eigenstates there. In other words, the bands on the k_x-axis can be labelled by their parity (odd or even) under M_y. Two bands with different parity are immune to level repulsion and they, thus, can cross leading to Dirac cones. The cones are robust because a perturbation that does not break M_y will slightly displace the crossing point but can not eliminate it. In the following we denote as $\mathbf{Q}_+(\mathbf{r})$ (even) and $\mathbf{Q}_-(\mathbf{r})$ (odd) the Bloch waves at the degeneracy point. By definition, we have

$$M_y \boldsymbol{Q}_\pm(\mathbf{r}) = \pm \boldsymbol{Q}_\pm(\mathbf{r}). \tag{3}$$

We note that M_y is the only unitary symmetry that maps one valley onto itself. The remaining unitary symmetries M_x and $M_x M_y$ (a π-rotation about the z-axis) map the two partner valleys one onto the other and, thus, do not directly constrain the form of the Dirac Hamiltonian for a fixed valley. However, they do it indirectly when combined with the time-reversal symmetry $\mathcal{T}$ to form the corresponding anti-unitary valley-preserving symmetries $\mathcal{T}M_x$ and $\mathcal{T}M_x M_y$. In the following, we can choose to focus on $\mathcal{T}M_x$ because a Hamiltonian that is invariant under M_y and $\mathcal{T}M_x$ will automatically be invariant under $\mathcal{T}M_x M_y$. With the goal of constraining the form of the Dirac Hamiltonian as much as possible, we fix (at least partially) the complex phase of $\boldsymbol{Q}_\pm(\mathbf{x})$ by requiring that

$$\mathcal{T}M_x \boldsymbol{Q}_\pm(\mathbf{x}) = \boldsymbol{Q}_\pm(\mathbf{x}). \tag{4}$$

We note that the above definition still allows to change the sign of $\boldsymbol{Q}_+(\mathbf{x})$ and/or $\boldsymbol{Q}_-(\mathbf{x})$. In view of performing a two band approximation we define the basis,

$$\boldsymbol{Q}_{A/B,\mathbf{p}}(\mathbf{r}) = e^{i\mathbf{p}\cdot\mathbf{r}} \boldsymbol{Q}_{A/B}(\mathbf{r}), \quad \boldsymbol{Q}_{A/B}(\mathbf{r}) = \frac{1}{\sqrt{2}}(\boldsymbol{Q}_+(\mathbf{r}) \pm \boldsymbol{Q}_-(\mathbf{r})). \tag{5}$$

where $\mathbf{p}$ is the quasi-momentum counted off from the degeneracy point. Note that by changing the sign of Q_+ or Q_- (but not both signs at the same time) will swap the labels A and B. We mention in passing that the Bloch waves $Q_{A,\mathbf{p}}$ and $Q_{B,\mathbf{p}}$ transform under the cmm group symmetries in the same way as plane waves on each of the two sublattices of graphene. This should facilitate the reading to readers familiar with the Dirac equation in this more well known context.

In view of finding the form of the local Hamiltonian, one needs preliminarily to derive how the basis $\boldsymbol{Q}_{A/B,\mathbf{p}}(\mathbf{r})$ transforms under the symmetry transformations M_y and $\mathcal{T}M_x$. From Eqs. (3,4,5), we find

$$\begin{aligned} M_y \boldsymbol{Q}_{A,(p_x,p_y)}(\mathbf{r}) &= \boldsymbol{Q}_{B,(p_x,-p_y)}(\mathbf{r}). \\ \mathcal{T}M_x \boldsymbol{Q}_{A/B,(p_x,p_y)}(\mathbf{r}) &= \boldsymbol{Q}_{A/B,(p_x,-p_y)}(\mathbf{r}). \end{aligned} \tag{6}$$

Next, we introduce a set of Pauli matrices $\hat{\sigma}_{i=x,y,z}$ such that $\hat{\sigma}_z$ is diagonal on the A/B basis and $\sigma_z = 1$ ($\sigma_z = -1$) for $\boldsymbol{Q}_{A,\mathbf{p}}(\mathbf{r})$ ($\boldsymbol{Q}_{B,\mathbf{p}}(\mathbf{r})$). From Eqs. (6) one, thus, see

$$M_y \hat{\sigma}_x M_y = \hat{\sigma}_x, \quad M_y \hat{\sigma}_{y/z} M_y = -\hat{\sigma}_{y/z}, \quad \mathcal{T}M_x \hat{\sigma}_{x/z} \mathcal{T}M_x = \hat{\sigma}_{x/z}, \quad \mathcal{T}M_x \hat{\sigma}_y \mathcal{T}M_x = -\hat{\sigma}_y. \tag{7}$$

while at the same time changing the quasi-momentum: under both M_y and $\mathcal{T}M_x$ we have

$$(p_x, p_y) \to (p_x, -p_y). \tag{8}$$

Thus, for example the interaction $p_x \hat{\sigma}_y$ will transform to $-p_x \hat{\sigma}_y$ under the mirror symmetry M_y because σ_y changes sign, cf. 7, while p_x remains invariant, cf. Eqs. 8. Using Eqs. (7) and (8) we can easily determine the form of the Dirac equation. We are interested in a (small) region about the degeneracy point and, thus, we can restrict ourselves to linear terms in the quasi-momentum. Considering all possible linear terms and keeping only those that are invariant under both M_y and $\mathcal{T}M_x$, we arrive at the massless Dirac Hamiltonian

$$H_D = \bar{\Omega} + (v_0 + v_x \hat{\sigma}_x) p_x + v_y \hat{\sigma}_y p_y. \tag{9}$$

This gives rise to gapless cones described by the band structure

$$\Omega_{e/g} = \bar{\Omega} + v_0 p_x \pm \sqrt{(v_x p_x)^2 + (v_y p_y)^2}. \tag{10}$$

Compared to the standard setting with $\mathcal{C}_3$ symmetry, here, the speed depends on the direction (because $v_x \neq v_y$). Moreover, in the same direction the speed is different for the ground and excited band (because $v_0 \neq 0$). Above the critical value of $|v_0|$, $|v_0| = |v_x|$, the band structure become qualitatively different leading to so-called type II Dirac cones [5]. For type II cones there is a direction where the speed is zero for one of the two bands. In the following, we do not discuss further this scenario as our experiment is in the regime where $|v_0| < |v_x|$.

Derivation of the full Dirac Hamiltonian within one domain

Next, we consider the case where the mirror symmetry M_y is broken but M_x is still a symmetry. Thus, the relevant Wallpaper group is now cm (point group $\mathcal{C}_\nu$). Using Eqs. (7) and (8) to find all possible terms that preserves the symmetry $\mathcal{T}M_x$, we arrive at the Dirac Hamiltonian

$$H_D = \bar{\Omega} + (v_0 + v_x \hat{\sigma}_x)\hat{p}_x + v_y \hat{\sigma}_y \hat{p}_y + (m + m'\hat{p}_x)\sigma_z. \tag{11}$$

This give rise to the band structure

$$\Omega_{e/g} = \bar{\Omega} + v_0 p_x \pm \sqrt{(m + m' p_x)^2 + (v_x p_x)^2 + (v_y p_y)^2}. \tag{12}$$

We note that within the expansion in $\mathbf{p}$ that underlies the Dirac Hamiltonian, we must assume $|m' p_x| \ll |m|$. Moreover, when the design with broken M_y-symmetry is obtained by modifying a design with conserved M_y symmetry (as in our case) all the parameters in Eq. (9) are renormalized, including the point on the k_x-axis from which the quasi-momentum is counted. We also note that for $p_x = p_y = 0$ in Eq. (11) the Bloch waves are eigenstates of σ_z. Since σ_z is by definition diagonal in the A/B basis from Eq. (6) it follows that for this particular quasi-momentum the Bloch waves are mirror-symmetric partners of each other. This feature can serve as a definition of the origin $p_x = 0$. This implies that the Bloch waves shown in Fig. 1(e-f) of the Main Text, which look very much like mirror-symmetric partners in the xz plane, have displaced quasi-momentum $\mathbf{p}$ very close to the origin. Once the point where $p_x = 0$ is known, we use Eq. (12) to fit the parameters in the Dirac Hamiltonian (the fitted values are given in the Main Text). We note that this way of fitting does not allow to fix the sign of v_x, v_y, and m. In particular, the sign of $m v_x v_y$ is still unknown. As we discuss below this sign fixes the value of the valley Chern number and to be able to determine it, it is not enough to examine the band structure alone but one rather needs to have information regarding the Bloch waves, see below.

Berry curvature and Valley Chern numbers

It is convenient to introduce the set of rotated Pauli matrices

$$\tau_x = \frac{v_x}{\sqrt{v_x^2 + m'^2}}\sigma_x + \frac{m'}{\sqrt{v_x^2 + m'^2}}\sigma_z, \quad \tau_y = \sigma_y, \quad \tau_z = \frac{v_x}{\sqrt{v_x^2 + m'^2}}\sigma_z - \frac{m'}{\sqrt{v_x^2 + m'^2}}\sigma_x. \tag{13}$$

In terms of the new set of Pauli matrices, the Dirac Hamiltonian Eq. (11) takes the simpler form

$$H_D = \bar{\Omega} + (v_0 + V_x \hat{\tau}_x)(\hat{p}_x - p_x^{(0)}) + V_y \hat{\tau}_y \hat{p}_y + M\tau_z, \tag{14}$$

$$V_x = (v_x^2 + m'^2)^{1/2}, \quad V_y = v_y, \quad M = \frac{m v_x}{V_x}, \quad p_x^{(0)} = -\frac{m m'}{V_x^2}. \tag{15}$$

The band structure in terms of the rescaled parameters reads

$$\Omega_{e/g} = \bar{\Omega} + v_0 p_x \pm \sqrt{M^2 + V_x^2 (p_x - p_x^{(0)})^2 + (V_y p_y)^2}. \tag{16}$$

Thus, $p_x^{(0)}$ is the bottom of the valley where the band splitting is minimum (when counted off from the quasi-momentum where the two Bloch waves are one the mirror symmetry of the other in the zx-plane) and M is the bulk band gap.

The Berry curvature for the lowest band of the Dirac Hamiltonian is (see [6] for the first equality)

$$\mathcal{B}(\mathbf{p}) = -2\mathrm{Im}\frac{\langle g,\mathbf{p}|\nabla_{p_x}H_D(\mathbf{p})|e,\mathbf{p}\rangle\langle e,\mathbf{p}|\nabla_{p_y}H_D(\mathbf{p})|g,\mathbf{p}\rangle}{(\Omega_e(\mathbf{p})-\Omega_g(\mathbf{p}))^2} = \frac{MV_xV_y}{2\left(M^2+V_x^2(p_x-p_x^{(0)})^2+(V_yp_y)^2\right)^{3/2}}, \tag{17}$$

where$|g,\mathbf{p}\rangle$ and $|e,\mathbf{p}\rangle$ are the ground and excited Bloch waves, respectively. As usual, the Chern number is defined as an integral of the Berry curvature [6]. For the valley Chern number, the integral over the BZ is replaced by an integral over the 2D plane,

$$C_v = -\frac{1}{2\pi}\int d^2\mathbf{p}\mathcal{B}(\mathbf{p}) = -\frac{1}{2}\mathrm{sgn}(mv_xv_y). \tag{18}$$

Limits of validity of the Dirac approach

The valley Chern numbers are well defined if the Berry curvature of the real bands is strongly peaked in an isolated region surrounded by a small Berry curvature region. In fact, it makes sense to identify each valley exactly with such isolated large Berry curvature region. The Berry curvature as calculated using the Dirac Hamiltonian is peaked in an ellipse-shaped region with axes M/V_x and M/V_y about $\mathbf{p}=(p_x^{(0)},0)$, cf. Eq. (17). We have to require that this region remains within the quasi-momentum region where the linear expansion leading to the Dirac equation is valid. The typical size of this region is $\sim 1/a_m$. We, thus, arrive to the condition,

$$M/a_m \ll V_x, V_y. \tag{19}$$

Identifying the valley Chern numbers from FEM simulations

Next, we want to determine the valley Chern number for our particular structure. We aim to use the formula $C_v = -\frac{1}{2}\mathrm{sgn}(mv_xv_y)$ and use some limited input from our FEM simulations to identify the sign of mv_xv_y. As discussed above our definitions Eqs. (3,4,5) have the disadvantage of not completely fixing the gauge in the Dirac equation: we have the freedom to identify either of the Bloch waves in Fig. 1(e,f) with $\boldsymbol{Q}_A$. Swapping the two states will have the effect of changing the sign of both m and v_y but will not change the gauge invariant quantity, C_v. Ideally we would like an alternative definition that completely fix the gauge. Such definition would, thus, determine which state should be identified with $\boldsymbol{Q}_A$. In this scenario, the sign of v_xv_y will be fixed by the gauge choice while the sign of m could be read out directly from the band structure and the symmetry of the Bloch waves for $\mathbf{p}=0$.

We can achieve exactly this if we view our system with space group cm (for the gapped cones) and cmm (for the gapless cones) as derived by the $\mathcal{C}_3$ symmetry breaking in a system with space group p3m1 (gapped cones) and p6m (gapless cones). In our particular case, the symmetry breaking comes from the silicon anisotropy and the elongated shape of the optical cavities. For the $\mathcal{C}_3$ symmetric case, we will use the $\mathcal{C}_3$ symmetry to fix the gauge and identify a robust feature to identify the normal mode $\boldsymbol{Q}_A$. Afterwards, in the symmetry broken case, we will appeal to a continuity argument to find the valley Chern number, see below.

The scenario with $\mathcal{C}_3$ symmetry is discussed in detail in [7]. In this case, we recover Eq. (11) with $v=v_x=v_y$ and $m', v_0 = 0$ if: (i) The quasi-momentum is counted off from the high-symmetry point $\mathbf{K}$. (ii) We identify the Bloch waves $\boldsymbol{Q}_{A/B}(\mathbf{r})$ with eigenstates of the $\mathcal{C}_3$ rotations with quasi-angular momentum m_c about the $\mathcal{C}_6$ rotocenter of the original $\mathcal{C}_{6\nu}$ symmetric design (the center of the snowflakes), $m_c=-\sigma_z$. (iii) We fix their phases to fulfill Eq. (6) [7]. By requiring $m_c=-\sigma_z$ we have fixed the sign of v_xv_y to be positive, thereby, there is no further ambiguity in the sign of m which now determines the valley Chern number, $C_v=-\mathrm{sign}(m)/2$. For our particular geometry it is useful to keep in mind that the p3m1 has three rotocenters. In our geometry, the two additional rotocenters lie at the centers of the upward and downward-pointing triangles. The Bloch waves $\boldsymbol{Q}_{A/B}(\mathbf{r})$ are simultaneous eigenstates of all rotations about any of the three rotocenters. The quasi-angular momentum $m_{d/u}$ for the rotations about the center of the downward/upward-pointing triangles is [7]

$$m_{d/u} = (m_c \pm 1 + 1)\mathrm{mod}3 - 1. \tag{20}$$

For the Bloch wave $\boldsymbol{Q}_A(\mathbf{r})$ we have $m_d=0$, which means that it displays a breathing motion in the downward-pointing triangles. Likewise, the mode $\boldsymbol{Q}_B(\mathbf{r})$ displays a breathing motion in the upper triangle.

Once the perturbation breaking the $\mathcal{C}_3$ symmetry is introduced the Bloch waves $\boldsymbol{Q}_{A/B}(\mathbf{r})$ are not anymore exact eigenstates of the $\mathcal{C}_3$ rotations, nevertheless, the breathing motion is still clearly visible, cf. Fig. 1 (e-f). This allows

us to identify the Bloch wave in Fig 1(f) (breathing motion in the downward-pointing triangles) with $\boldsymbol{Q}_A(\mathbf{r})$ with the expectation that the product $v_x v_y$ will be positive (the velocities should remain similar as in the $\mathcal{C}_3$ symmetric limit, $v_x, v_y \sim v$). Moreover, from the band structure Fig. 1(d) we see that the mass m is positive. We can conclude that the valley Chern number is $C_v = -1/2$ for domain 1, cf. Eq. (18).

Derivation of the Dirac equation in a system combining domain 1 and domain 2

If the parameters of Eq. (11) for domain 1 are known one can easily find the parameters for domain 2 (which is the mirror image of domain 1 in the zx plane) by transforming Eq. (11) under the mirror symmetry M_y. Using Eqs. (7) and (8), we see that v_0, v_x, and v_y remain the same in the two domains while m and m' change sign. Thus, the valley Chern number, cf. Eq. (18), changes sign in domain 2, $C_v = 1/2$.

In a system combining both domain 1 and domain 2 we assume that a normal mode $\mathbf{Q}_n(\mathbf{r})$ can be obtained by multiplying the Bloch waves Eq. (5) for $\mathbf{p} = 0$ by a smooth envelope,

$$\mathbf{Q}_n(\mathbf{r}) = \psi_{n,A}(\mathbf{r})\mathbf{Q}_A(\mathbf{r}) + \psi_{n,B}(\mathbf{r})\mathbf{Q}_B(\mathbf{r}). \tag{21}$$

This leads to the time-independent Schroedinger equation

$$\Omega_n \boldsymbol{\psi}_n(\mathbf{r}) = \hat{H}_D \boldsymbol{\psi}_n(\mathbf{r}), \tag{22}$$

where $\boldsymbol{\psi}_n(\mathbf{r})$ groups the smooth envelopes in a vector, $\boldsymbol{\psi}_n(\mathbf{r}) = (\psi_{n,A}(\mathbf{r}), \psi_{n,B}(\mathbf{r}))$, and $\hat{H}_D$ is the Dirac Hamiltonian Eq. (1) of the Main Text

$$\hat{H}_D = \bar{\Omega} + (v_0 + v_x\hat{\sigma}_x)\hat{p}_x + v_y\hat{\sigma}_y\hat{p}_y + \{\Theta(\hat{\mathbf{r}}), (m + m'\hat{p}_x)\}\hat{\sigma}_z. \tag{23}$$

Here $\Theta(\mathbf{r}) = 1/2$ ($\Theta(\mathbf{r}) = -1/2$) in domain 1 (2). We note that, here, position and quasi-momentum are non-commuting operators, thus, requiring the introduction of the anti-commutator $\{,\}$ to make sure that the Hamiltonian is hermitian.

Solution of the Dirac equation for a strip configuration

Next, we look for gapless eigenstates of Hamiltonian Eq. (23) in the presence of translationally invariant domain walls. In this scenario, the quasi-momentum in the translationally invariant direction is a conserved quantity and the smooth envelope depends only on the coordinate transverse to the domain wall.

Edge states for a horizontal strip.

For a horizontal strip, the displacement field for smooth envelope Bloch waves takes the form

$$\mathbf{Q}_{p_x}(\mathbf{r}) = \psi_{A,p_x}(y)e^{ip_x x}\mathbf{Q}_A(\mathbf{r}) + \psi_{B,p_x}(y)e^{ip_x x}\mathbf{Q}_B(\mathbf{r}). \tag{24}$$

This results in a Hamiltonian of the form

$$\hat{H}_D = \bar{\Omega} + (v_0 + v_x\hat{\sigma}_x)p_x - iv_y\hat{\sigma}_y\frac{d}{dy} + 2\Theta(y)\,(m + m'p_x)\,\hat{\sigma}_z. \tag{25}$$

We are interested in the same configuration as in Fig 1(g) of the Main Text where domain 1 (domain 2) is in the lower-half (upper-half) plane. This choice corresponds to $\Theta(y) = 1/2$ for $y < 0$, and $\Theta(y) = -1/2$ otherwise. Until now we have assumed $m > 0$. We note that if we keep $\Theta(y)$ fixed, and changing the sign of both m and m' describes a scenario where the two domains are swapped. In the following, we want to compare these two scenarios. For this purpose, we look for gapless edge eigenstates of the Hamiltonian Eq. (25) without committing on the sign of m and m'. We find

$$\boldsymbol{\psi}_{p_x}(y) = e^{-|(m+m'p_x)y/v_y|}\begin{pmatrix} 1 \\ \mathrm{sign}(v_y m)\end{pmatrix}, \quad \Omega_{p_x} = \bar{\Omega} + (\mathrm{sign}(v_y m)v_x + v_0)p_x. \tag{26}$$

This solution is valid for $|m| > |m'p_x|$ (within the limit of validity of the linear expansion that underlies the Dirac equation). In the following, we also assume that $|v_x| > |v_0|$ (in the scenario where this condition is violated the Dirac

cones are of type II and the mass term does not lead to a global band gap.) From Eq. (26) we see that, as expected, the propagation direction changes when the domains are swapped according to the bulk boundary correspondence [6]. More precisely, the edge state is a right mover if domain 1 is in the lower-half plane (for $m > 0$, $v_x v_y > 0$). Vice versa, it is a left mover if domain 1 is in the upper-half plane (for $m < 0$, $v_x v_y > 0$). This is consistent with the bulk-boundary correspondence because the valley Chern number C_v is $-1/2$ in domain 1 and $1/2$ in domain 2 and, thus, the edge state is a right (left) mover if the Chern number increases (decreases) by one across the domain wall. This is also in agreement with our FEM simulations, cf. Fig1(i, left panel) of the Main Text where the Dirac cone close to the **K**-point which has positive quasi-momentum k_x in the first Brillouin zone of the bulk has negative quasi-momentum in the first Brillouin zone of a horizontal strip.

Until now, we have discussed general features of the Valley Hall effect that are not unique to our setting with broken $\mathcal{C}_3$ symmetry. Eq. (26) predicts also other more surprising features that are unique to our setting. Most remarkably, the speed $|v_x + \text{sign}(m) v_0|$ changes if the two domains are swapped (changing the sign of m.) Moreover, the localization length of the edge state depends on the longitudinal quasi-momentum p_x. Both these features are confirmed in FEM simulations of a strip with a horizontal domain wall.

Edge states for a slanted strip.

Next, we calculate the solution when the domain wall is along the line $\tilde{y} = -y/2 + \sqrt{3}x/2 = 0$ (240° strip). In this scenario, it is convenient to change to a rotated frame with coordinates $\tilde{y}$ (transverse to the domain wall) and $\tilde{x} = -x/2 - \sqrt{3}y/2$ (longitudinal to the domain wall). In this case, the conserved quasi-momentum is $p_{\tilde{x}}$ (in the direction $\tilde{x}$) and the envelope is a function of $\tilde{y}$, $\boldsymbol{\psi}_{p_{\tilde{x}}}(\tilde{y})$. The Dirac Hamiltonian in terms of the rotated quasi-momenta and Pauli matrices,

$$p_x = -p_{\tilde{x}}/2 + \sqrt{3}p_{\tilde{y}}/2, \quad p_y = -p_{\tilde{y}}/2 - \sqrt{3}p_{\tilde{x}}/2,$$
$$\sigma_x = -\sigma_{\tilde{x}}/2 + \sqrt{3}\sigma_{\tilde{y}}/2, \quad \sigma_y = -\sigma_{\tilde{y}}/2 - \sqrt{3}\sigma_{\tilde{x}}/2,$$

reads

$$\hat{H}_D = -i\partial_{\tilde{y}} \left(h'_0 + h'_{\tilde{x}}\sigma_{\tilde{x}} + h'_{\tilde{y}}\sigma_{\tilde{y}}\right) + \{-i\partial_{\tilde{y}}, \Theta(\tilde{y})\} h'_z \hat{\sigma}_z + h_0 + h_{\tilde{x}}\sigma_{\tilde{x}} + h_{\tilde{y}}\sigma_{\tilde{y}} + 2\Theta(\tilde{y}) h_z \sigma_z \tag{27}$$

where

$$h'_0 = \frac{\sqrt{3}}{2}v_0, \quad h'_{\tilde{x}} = \frac{\sqrt{3}}{4}(v_y - v_x), \quad h'_{\tilde{y}} = \frac{1}{4}(v_y + 3v_x), \quad h'_z = \frac{\sqrt{3}}{2}m'$$
$$h_0 = \bar{\Omega} - \frac{v_0}{2}p_{\tilde{x}}, \quad h_{\tilde{x}} = \frac{1}{4}\left(v_x + 3v_y\right)p_{\tilde{x}}, \quad h_{\tilde{y}} = \frac{\sqrt{3}}{4}(v_y - v_x)p_{\tilde{x}}, \quad h_z = m - \frac{1}{2}m' p_{\tilde{x}}.$$

In this case, to solve the Dirac equation (22) with the Dirac Hamiltonian in the more general form Eq. (27) we have to use the ansatz,

$$\boldsymbol{\psi}_{p_{\tilde{x}}}(\tilde{y}) = (1 + i\epsilon\Theta(\tilde{y}))e^{-|\tilde{y}|/\xi + i\Xi\tilde{y}} \begin{pmatrix} 1 \\ b \end{pmatrix}. \tag{28}$$

Compared to Eq. (26) this more general ansatz allows for oscillations of the wave function with period $2\pi/\Xi$. By plugging the anstaz Eq. (28) into the Dirac equation (22), we find three types of terms: (i) terms containing a delta function, (ii) terms in the form $M_i\Theta(\tilde{y})\psi_{p_{\tilde{x}}}(\tilde{y})$ where M_i are matrices independent of $\tilde{y}$, and (iii) terms that depends on $\tilde{y}$ only via the wave function $\boldsymbol{\psi}_{p_{\tilde{x}}}(\tilde{y})$. By isolating the terms proportional to $\delta(\tilde{y})$ and requiring their sum to be zero, we find the equation

$$\left[ih'_z\sigma_z + \epsilon\left(h'_0 + h'_{\tilde{x}}\sigma_{\tilde{x}} + h'_{\tilde{y}}\sigma_{\tilde{y}}\right)\right]\begin{pmatrix} 1 \\ b \end{pmatrix} = 0. \tag{29}$$

It has a solution if

$$\det\left[ih'_z\sigma_z + \epsilon\left(h'_0 + h'_{\tilde{x}}\sigma_{\tilde{x}} + h'_{\tilde{y}}\sigma_{\tilde{y}}\right)\right] = 0. \tag{30}$$

One immediately finds two possible solutions

$$\epsilon_{\pm} = \pm\frac{h'_z}{\sqrt{h'^2_{\tilde{x}} + h'^2_{\tilde{y}} - h'^2_0}}. \tag{31}$$

The corresponding b is

$$b_\pm = -\frac{h'_0 h'_{\tilde{x}} \mp \sqrt{h'^2_{\tilde{x}} + h'^2_{\tilde{y}} - h'^2_0}\, h'_{\tilde{y}} + i(h'_0 h'_{\tilde{y}} \pm \sqrt{h'^2_{\tilde{x}} + h'^2_{\tilde{y}} - h'^2_0}\, h'_{\tilde{x}})}{h'^2_{\tilde{x}} + h'^2_{\tilde{y}}}. \tag{32}$$

Note that $|b_\pm| = 1$ and, thus, the vectors $(1, b_\pm)^T$ lie on the equator of the Bloch sphere. Next, we require that the sum of the terms in the form $\propto \Theta(\tilde{y})\boldsymbol{\psi}_{p_{\tilde{x}}}(\tilde{y})$ is zero. We find the equation

$$\left[\xi(h_z + h'_z \Xi)\sigma_z + i\left(h'_0 + h'_{\tilde{x}}\sigma_{\tilde{x}} + h'_{\tilde{y}}\sigma_{\tilde{y}}\right)\right]\begin{pmatrix}1\\ b\end{pmatrix}. \tag{33}$$

where b should be equal either to b_+ or b_-. By requiring that the determinant is zero we find

$$\xi = \frac{1}{|h_z + h'_z \Xi|}\left(h'^2_{\tilde{x}} + h'^2_{\tilde{y}} - h'^2_0\right)^{1/2}. \tag{34}$$

By solving for b we find $b = b_+$ ($b = b_-$) if $h_z + h'_z\Xi < 0$ ($h_z + h'_z\Xi > 0$) independent of Ξ. Next, we need to require that the sum of the terms in the form $\propto \boldsymbol{\psi}_{p_{\tilde{x}}}(\tilde{y})$ is zero. We find the equation

$$\left(i\tilde{h}_z\sigma_z + \tilde{h}_{\tilde{x}}\sigma_{\tilde{x}} + \tilde{h}_{\tilde{y}}\sigma_{\tilde{y}} + \tilde{h}_0\right)\begin{pmatrix}1\\ b\end{pmatrix} = 0, \tag{35}$$

where

$$\tilde{h}_{\tilde{x}} = h_{\tilde{x}} + \Xi h'_{\tilde{x}}, \quad \tilde{h}_{\tilde{y}} = h_{\tilde{y}} + \Xi h'_{\tilde{y}}, \quad \tilde{h}_z = \frac{|h_z + h'_z\Xi|}{\left(h'^2_{\tilde{x}} + h'^2_{\tilde{y}} - h'^2_0\right)^{1/2}} h'_z, \quad \tilde{h}_0 = h_0 + \Xi h'_0 - \Omega_{p_{\tilde{x}}}. \tag{36}$$

To solve this it is convenient to define

$$\tilde{\phi} = \arg b, \quad \tilde{\sigma}_{\tilde{x}} = \cos\phi\sigma_{\tilde{x}} + \sin\tilde{\phi}\sigma_{\tilde{y}}, \quad \tilde{\sigma}_{\tilde{y}} = \cos\tilde{\phi}\sigma_{\tilde{y}} - \sin\tilde{\phi}\sigma_{\tilde{x}}. \tag{37}$$

and rewrite the equation in terms of the Pauli matrices $\tilde{\sigma}_{\tilde{x}}$ and $\tilde{\sigma}_{\tilde{y}}$,

$$\left(i\tilde{h}_z\sigma_z + (\cos\tilde{\phi}\tilde{h}_{\tilde{x}} + \sin\tilde{\phi}\tilde{h}_{\tilde{y}})\tilde{\sigma}_{\tilde{x}} + (\cos\tilde{\phi}\tilde{h}_{\tilde{y}} - \sin\tilde{\phi}\tilde{h}_{\tilde{x}})\tilde{\sigma}_{\tilde{y}} + \tilde{h}_0\right)\begin{pmatrix}1\\ b\end{pmatrix} = 0. \tag{38}$$

From Eq. (32), we can read out

$$\cos\tilde{\phi} = -\frac{h'_0 h'_{\tilde{x}} \pm \sqrt{h'^2_{\tilde{x}} + h'^2_{\tilde{y}} - h'^2_0}\, h'_{\tilde{y}}}{h'^2_{\tilde{x}} + h'^2_{\tilde{y}}}, \quad \sin\tilde{\phi} = -\frac{h'_0 h'_{\tilde{y}} \mp \sqrt{h'^2_{\tilde{x}} + h'^2_{\tilde{y}} - h'^2_0}\, h'_{\tilde{x}}}{h'^2_{\tilde{x}} + h'^2_{\tilde{y}}}. \tag{39}$$

We note that $\tilde{\phi}$ is independent of Ξ and p_x and that by construction

$$\tilde{\sigma}_{\tilde{x}}\begin{pmatrix}1\\ b\end{pmatrix} = \begin{pmatrix}1\\ b\end{pmatrix}, \quad \sigma_z\begin{pmatrix}1\\ b\end{pmatrix} = i\tilde{\sigma}_{\tilde{y}}\begin{pmatrix}1\\ b\end{pmatrix} = \begin{pmatrix}1\\ -b\end{pmatrix}. \tag{40}$$

Plugging the above relations into Eq. (38), we immediately find

$$\tilde{h}_z - \tilde{h}_{\tilde{y}}\cos\tilde{\phi} + \tilde{h}_{\tilde{x}}\sin\tilde{\phi} = 0, \quad \tilde{h}_{\tilde{x}}\cos\tilde{\phi} + \tilde{h}_{\tilde{y}}\sin\tilde{\phi} + \tilde{h}_0 = 0. \tag{41}$$

From the second equation we find

$$\Omega_{p_{\tilde{x}}} = h_0 + \Xi h'_0 + (h_{\tilde{x}} + \Xi h'_{\tilde{x}})\cos\tilde{\phi} + (h_{\tilde{y}} + \Xi h'_{\tilde{y}})\sin\tilde{\phi} \tag{42}$$

where Ξ is obtained by solving the first equation (which is a simple linear equation). The full expression for Ξ and $\bar{\Omega}$ is very cumbersome and does not give much physical insight and, thus, we omit it here. Instead, it is interesting to comment on its leading order expansion in $m'/\bar{v}, v_0/\bar{v}, \delta v/\bar{v}$ ($\bar{v} = (v_x + v_y)/2, \delta v = v_x - v_y$),

$$\Xi \approx \frac{\sqrt{3}m}{2\bar{v}}\frac{m'}{\bar{v}} + \frac{\sqrt{3}}{2}\left(-\mathrm{sign}(m\bar{v})\frac{v_0}{\bar{v}} + \frac{\delta v}{\bar{v}}\right)p_x, \tag{43}$$

$$\Omega_{p_{\tilde{x}}} \approx \bar{\Omega} + \bar{v}\left(\mathrm{sign}(m\bar{v}) - \frac{v_0}{2\bar{v}} - \mathrm{sign}(m\bar{v})\frac{\delta v}{4\bar{v}}\right)p_x. \tag{44}$$

From this expression we see that again the edge state (for the valley close to the **K** point) is a right mover if the domain 1 is in the lower $\tilde{y}$-plane (for $m > 0$). Also in this case (as for the horizontal domain wall) the speed changes if the two domains are exchanged. Compared to the horizontal domain wall, the edge state amplitude does not only decay away from the domain wall but it also displays oscillations with period $2\pi/\Xi$.

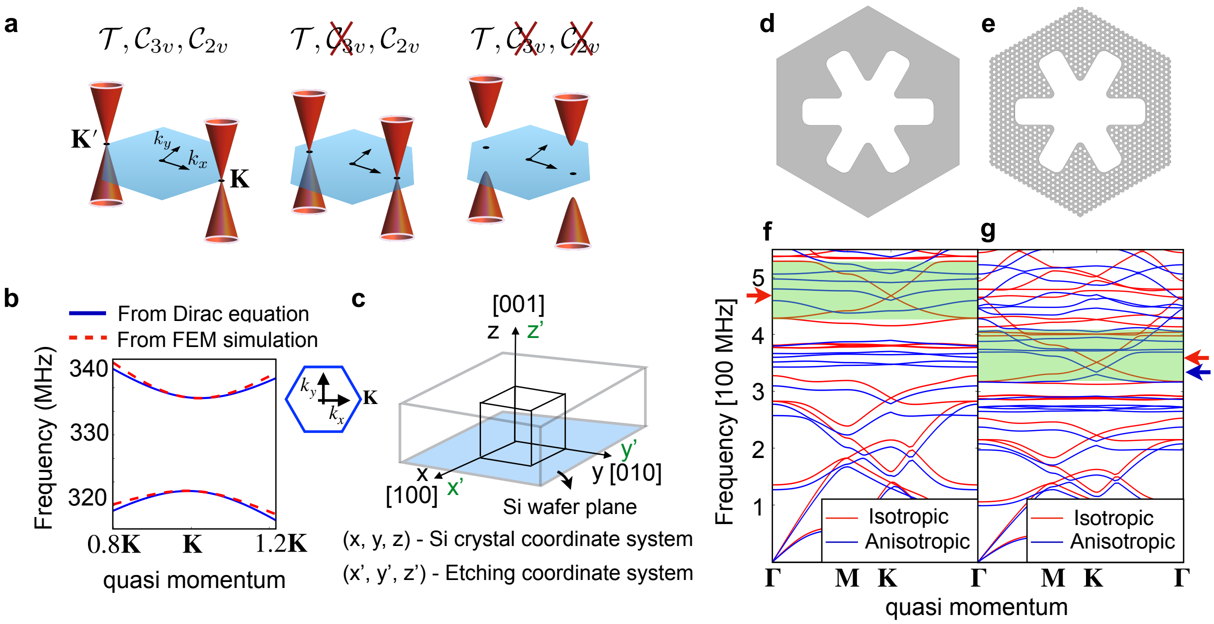

Supplementary Figure 2. Effect of symmetries and anisotropic material properties of Silicon on the Dirac band dispersion. **a**, Effects of different symmetries on the Dirac cones. Dirac cones are displaced from the K point when the $\mathcal{D}_{6v}$ symmetry (60-degree rotations and mirror symmetry) is broken. The degenerate cones split up when the $\mathcal{D}_{2v}$ symmetry is also broken. **b**, Comparison of the dispersion for the gapped Dirac cones, calculated from FEM simulations and the Dirac equation. **c**, The (x',y',z') axes of the fabricated devices are oriented with the [100], [010] and [001] planes of the Silicon cubic crystal, respectively. **d** and **e**, Unit cell geometry of the snowflake crystal in the presence or absence of the circular photonic crystal holes. **f** and **g**, Corresponding simulated phononic band structures with isotropic and anisotropic Silicon material properties respectively. Note that only the modes which are even under the mirror operator M_z ($z \mapsto -z$) are shown here. Odd modes do not couple to the light. The arrows indicate the Dirac cone frequencies. The Dirac cones are significantly distorted for the snowflake crystals without photonic crystal holes (d and f), compared to the one with photonic crystal holes (e and g).

Supplementary Note 5. EFFECT OF ANISOTROPIC MATERIAL PROPERTIES OF SILICON

The Silicon crystal structure has a cubic primitive lattice, which leads to anisotropic material properties, and thus, the phononic band structures of our phononic crystal devices depend on the orientation of alignment during fabrication with respect to the Silicon crystal. The surface plane of the silicon wafer we used is parallel to the (001) crystal plane of Silicon. The alignment co-ordinate axes (x',y',z') during fabrication are oriented to the axes ([100], [010], [001]) of the Silicon wafer, cf. Supplementary Figure 2c. Our FEM simulations take fully into account the anisotropy using an appropriate anisotropic elastic tensor [8]. In the presence of discrete translational symmetry, we may express the solutions to the acoustic wave equation as Bloch modes and numerically solve for the phononic band structure with an FEM solver [1]. Periodic boundary conditions are used to solve for the desired number of bands at any desired point within the Brillouin zone (BZ). The simulated phononic band structures for the two-dimensional snowflake unit cell are shown in Supplementary Figure 2f and g. We investigate the role of anisotropic material properties on the Dirac band dispersion. This is done by comparing the two-dimensional snowflake unit cell phononic band structure without (see Supplementary Figure 2d) and with (see Supplementary Figure 2e) circular photonic crystal holes for the two cases of isotropic (red solid lines) and anisotropic (blue solid lines) Silicon elasticity tensors. We notice that the Dirac bands for snowflakes without optical cavity holes (see Supplementary Figure 2f) are significantly distorted for the anisotropic silicon elasticity tensor. Therefore, the small circular photonic crystal holes counter the distortion of Dirac bands by the anisotropic silicon (see Supplementary Figure 2g).

Supplementary Note 6. OPTICAL READOUT OF THE THERMAL MECHANICAL POWER SPECTRUM

We measure the local thermal power spectrum of the mechanical modes in the bulk and domain wall regions. Readout of the mechanics is performed by observing the transduced sidebands in the optical cavity spectrum. We

strongly drive an optical cavity at a blue-detuning of $\bar{\Omega}$, corresponding to the middle of the mechanical bulk band gap. In the frame rotating with the laser frequency ω_L, the optical cavity (annihilation operator a) interacting with the mechanical normal modes (annihilation operator b_n for the n-th mode) is described by the set of coupled Langevin equations,

$$\dot{a} = \left(i\Delta - \frac{\kappa_t}{2}\right) a + i\sum_n g_{0n} a \left(b_n + b_n^\dagger\right) + \sqrt{\frac{\kappa_e}{2}} a_{in} \tag{45}$$

$$\dot{b}_n = \left(-i\Omega_n - \frac{\Gamma_n}{2}\right) b_n + i g_{0n} a^\dagger a + \sqrt{\Gamma_n} b_{in,n}$$

Here, $\Delta = \omega_L - \omega_{cav} = \bar{\Omega}$ is the detuning and $\kappa_t = \kappa_i + \kappa_e$ is the total optical decay rate. (We have a double-sided fiber taper coupling, because of which we observe only 50% of the output photons from the cavity.) The input phonon noise annihilation operator $b_{in,n}$ represents the interaction of the mechanical system with the thermal bath. The optical noise is negligible. The mechanical decay rates Γ_n are almost constant, $\Gamma_n/2\pi \approx 200\text{kHz}$, and we do not seek to model them. On the other hand, we determine the mechanical eigenfrequencies Ω_n and the single-photon optomechanical coupling rates g_{0n} combining FEM simulation of a strip configuration to scattering matrix calculations, see Supplementary Note Supplementary Note 7.

We linearize the equations of motion about the static equilibrium [9], and set $a = \alpha + \delta a e^{i\theta}$, where $\alpha = |\alpha| e^{i\theta}$ ($|\alpha|^2$ is the number of intra-cavity photons), and $b_n = \beta_n + \delta b_n$, with

$$\beta_n = \frac{i g_{0n} |\alpha|^2}{i\Omega_n + \Gamma_n/2} \approx \frac{g_{0n}|\alpha|^2}{\Omega_n} \tag{46}$$

$$\alpha = \frac{-\sqrt{\kappa_e/2} a_{in}}{i\Delta - \kappa_t/2 + i\sum_n g_{0n}(\beta_n + \beta_n^*)} \approx \frac{-\sqrt{\kappa_e/2} a_{in}}{i\Delta - \kappa_t/2 + 2i|\alpha|^2 \sum_n g_{0n}^2/\Omega_n} \approx \frac{-\sqrt{\kappa_e/2} a_{in}}{i\Delta - \kappa_t/2} \tag{47}$$

Here, we have used $\Omega_j \gg \Gamma_j$ and $\kappa_t \gg |\alpha|^2 \sum_n g_{0n}^2/\Omega_n$. Ignoring the nonlinear interaction, the resulting equation of motion is

$$\delta\dot{a} = \left(i\bar{\Delta} - \frac{\kappa_t}{2}\right) \delta a + i\sum_n g_{0n}|\alpha| \left(\delta b_n + \delta b_n^\dagger\right) \tag{48}$$

$$\delta\dot{b}_n = \left(-i\Omega_n - \frac{\Gamma_n}{2}\right) \delta b_n + i g_{0n}|\alpha| \left(\delta a + \delta a^\dagger\right) + \sqrt{\Gamma_n} b_{in,n} \tag{49}$$

Here, $\bar{\Delta} = \Delta + \sum_n g_{0n}\left(\beta_n + \beta_n^*\right)$. For the frequency domain operators defined by $O[\omega] = \frac{1}{2\pi}\int_{-\infty}^{+\infty} d\omega e^{i\omega t} O(t)$ and $O^\dagger[\omega] = \frac{1}{2\pi}\int_{-\infty}^{+\infty} d\omega e^{i\omega t} O^\dagger(t) = [O[-\omega]]^\dagger$, the above equation can be recasted to the following linear system of algebraic equations

$$\delta a[\omega] = i|\alpha| \chi_{opt}[\omega] \sum_n g_{0n} \left(\delta b_n[\omega] + \delta b_n^\dagger[\omega]\right) \tag{50}$$

$$\chi_n^{-1}[\omega]\delta b_n[\omega] = \sqrt{\Gamma_n} b_{in,n}[\omega] - i|\alpha|^2 g_{0n} \left(\chi_{opt}[\omega] - \chi_{opt}^*[-\omega]\right) \sum_{n'} g_{0n'} \left(\delta b_{n'}[\omega] + \delta b_{n'}^\dagger[\omega]\right) \tag{51}$$

$$\left(\chi_n^{-1}[-\omega]\right)^* \delta b_n^\dagger[\omega] = \sqrt{\Gamma_n} b_{in,n}^\dagger[\omega] - i|\alpha|^2 g_{0n} \left(\chi_{opt}[\omega] - \chi_{opt}^*[-\omega]\right) \sum_{n'} g_{0n'} \left(\delta b_{n'}[\omega] + \delta b_{n'}^\dagger[\omega]\right) \tag{52}$$

where $\chi_n[\omega] = \left[\Gamma_n/2 - i\left(\omega - \Omega_n\right)\right]^{-1}$ and $\chi_{opt}[\omega] = \left[\kappa_t/2 - i\left(\omega + \bar{\Delta}\right)\right]^{-1}$ are the mechanical and optical susceptibilities, respectively, in the absence of optomechanical coupling. We can ignore the optical backaction on the mechanics because our experiment is in a regime of small optomechanical cooperativity. Thus,

$$\delta a[\omega] = i|\alpha|\chi_{opt}[\omega] \sum_n g_{0n}\sqrt{\Gamma_n} \left(\chi_n[\omega] b_{in,n}[\omega] + \left(\chi_n[-\omega]\right)^* b_{in,n}^\dagger[\omega]\right) \tag{53}$$

The output from the cavity is obtained via the input-output relations as

$$a_{out}[\omega] = a_{in} - \sqrt{\frac{\kappa_e}{2}} \left(\alpha + \delta a[\omega] e^{i\theta}\right) \tag{54}$$

$$= a_{in}\frac{i\Delta - \kappa_i/2}{i\Delta - \kappa_t/2} + a_{in}\frac{\kappa_e/2}{i\Delta - \kappa_t/2} i\chi_{opt}[\omega] \sum_n g_{0n}\sqrt{\Gamma_n} \left(\chi_n[\omega] b_{in,n}[\omega] + \left(\chi_n[-\omega]\right)^* b_{in,n}^\dagger[\omega]\right) = \bar{a}_{out} + \delta a_{out}[\omega] \tag{55}$$

In our detection scheme, the strong laser beats with the optical sidebands. This generates photo-current proportional to the cavity output amplitude quadrature $I(t) = \delta a_{out}(t) + \delta a^{\dagger}_{out}(t)$. The cavity output power spectrum density is given by

$$S_{\rm II}[\omega] = \int_{-\infty}^{\infty} dt e^{i\omega t} \langle I(t)I(0)\rangle = 2\pi \int_{-\infty}^{\infty} d\omega' \langle I[\omega]I[\omega']\rangle \tag{56}$$

For a thermal bath of average phonon occupancy n_b, the correlation of the noise operators are $\left\langle b^{\dagger}_{in,n}[\omega] b_{in,k}[\omega']\right\rangle = n_b/(2\pi)\delta(\omega+\omega')\delta_{n,k}$ and $\left\langle b_{in,n}[\omega] b^{\dagger}_{in,k}[\omega']\right\rangle = (n_b+1)/(2\pi)\delta(\omega+\omega')\delta_{n,k}$. At room temperature $T = 300K$ and $\Omega_n = 330$MHz, $n_b \approx k_B T/\hbar\Omega_n \approx 18940$ phonons. The boson occupancy is practically identical for all the standing wave mechanical normal modes, and we also assume $n_b + 1 \approx n_b$ for simplicity. For $\omega \approx \bar{\Omega}$ and $\Omega_n \gg \Gamma_n$, we find

$$S_{\rm II}[\omega] = \frac{\kappa_e}{4}\left|\alpha\chi_{opt}[\omega] - \alpha^*\chi^*_{opt}[-\omega]\right|^2 \sum_n \frac{2g_{0n}^2 n_b \Gamma_n}{(\omega-\Omega_n)^2 + (\Gamma_n/2)^2} = \frac{\kappa_e}{4}\left|\alpha\chi_{opt}[\omega] - \alpha^*\chi^*_{opt}[-\omega]\right|^2 \sum_n G_n^2 S_{{\rm mech},n}[\omega] \tag{57}$$

$$G_n = \frac{g_{0n}}{x_{zpf,n}},\ S_{{\rm mech},n}[\omega] = \frac{2x^2_{zpf,n} n_b \Gamma_n}{(\omega-\Omega_n)^2 + (\Gamma_n/2)^2}, \tag{58}$$

where $x_{zpf,n}$ are the zero point fluctuations of the n-th mode.

So far the discussion has been generic and could refer to any cavity coupled to multiple mechanical modes. Next, we discuss the particular features that arise in the position resolved noise spectrum of our topological mechanical cavity because of the underlying topology. As discussed in the Main Text, the spectrum Ω_j in the topological region is formed by a series of quasidegenerate doublets because of the suppression of backscattering (see the derivation below based on the scattering matrix approach). Each of these modes is a standing wave mode leading to a dependence of the optomechanical coupling on the optical cavity via its position (alternatively, as long as they are quasi-degenerate, they can be treated as running waves that are counterpropagating, leading to the same final result displayed below). Away from a corner, the domain wall hosting the topological mechanical cavity can be approximated as a 1D strip, and we expect the coupling to display a sinusoidal dependence on the cavity position, $G_n(j) \propto \cos(k_l(\Omega_j) j a_m + \phi_j)$ where a_m is the length of strip unit cell, ja_m is the position of the j-th cavity and $k_l(\Omega_n)$ is the quasi-momentum (which depends on the direction of the domain wall l), see below for derivation. For quasidegenerate levels ($\Omega_n \approx \Omega_{n+1}$), the couplings $G_n(j)$ and $G_{n+1}(j)$ will then be sinusoidal waves with the same period but a phase delay $\phi_{n+1} - \phi_n \approx \pi/2$. From Eq. (57) we see that two quasidegenerate levels with a splitting $\Omega_n - \Omega_{n+1}$, much smaller than the mechanical decay rate Γ_n, give rise to a single mechanical noise spectrum peak with a height proportional to the sum of the squares of their respective optomechanical couplings,

$$S_{\rm II}[\Omega_n] \propto G_n^2 + G_{n+1}^2 \approx \cos^2(k_l(\Omega_n) j a_m + \phi_n) + \sin^2(k_l(\Omega_n) j a_m + \phi_n) \approx {\rm const.} \tag{59}$$

Thus, we find that the peak height is the same for all cavities that are localized along the same edge of the polygonal-shaped domain wall and away from the corners. This feature is visible in the measured position resolved spectrum shown in Fig. 2(d) of the Main Text.

Supplementary Note 7. SEMI-ANALYTICAL CALCULATION OF THE SPECTRA AND OPTOMECHANICAL COUPLING FOR THE MECHANICAL TOPOLOGICAL CAVITY

Here, we discuss how we calculate the mechanical eigenfrequencies Ω_j and optomechanical couplings g_{0j} used for the theoretical estimation of the power spectra of the mechanical topological cavity. A full FEM simulation of our device is not feasible due to the multiscale nature of our optomechanical crystal and the large system size. Instead, we adopt a hybrid approach where the spectra and optomechanical couplings are obtained from a scattering matrix calculation that uses the band structure and optomechanical couplings of strip configurations (with the two relevant orientations of the domain wall) obtained using FEM simulation as input.

Scattering matrix calculation of the spectrum Ω_n

We model the topological mechanical cavity as a closed sequence of edge channels connected by scattering centers. We assume that away from the corners the mechanical waves propagate unimpeded as if they were flowing in an infinitely long edge channel. On the other hand, we describe the transmission across the corners phenomenologically

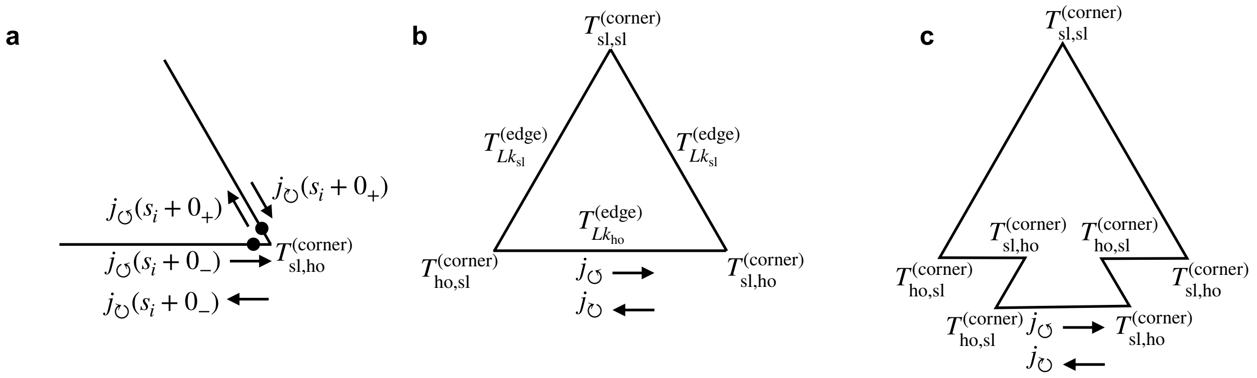

Supplementary Figure 3. **Schematic for the one-dimensional scattering matrix model.** **a**, Wave transport from the horizontal to the slanted waveguide through the corner is modelled with the transfer matrix $T^{(\mathrm{corner})}_{\mathrm{sl,ho}}$. **b** (**c**), Modelling wave transport on the triangular (tree) topological mechanical cavities with different transfer matrices.

using the standard scattering-matrix approach. In the following, we assume that the domain 1 region has a polygonal form with sides in the horizontal direction (along the x-axis) or two different slanted directions (120° or 240° from the x-axis). This general scenario applies to all the devices we have built.

We introduce the coordinate s that follows the domain wall length and denote as $j_{\circlearrowright}(s)$ and $j_{\circlearrowleft}(s)$ the mechanical edge current amplitudes circulating clockwise and anti-clockwise about domain 2, respectively (thus, $|j_{\circlearrowright}(s)|^2$ and $|j_{\circlearrowleft}(s)|^2$ are the corresponding mechanical energy fluxes).

Away from a corner, we assume that the mechanical wave propagates as in an infinitely long strip,

$$\begin{pmatrix} j_{\circlearrowright}(s+na_m) \\ j_{\circlearrowleft}(s+na_m) \end{pmatrix} = T^{(\mathrm{edge})}_{na_m k_l(\Omega)} \begin{pmatrix} j_{\circlearrowright}(s) \\ j_{\circlearrowleft}(s) \end{pmatrix}, \quad T^{(\mathrm{edge})}_{\theta} = \begin{pmatrix} e^{-i\theta} & 0 \\ 0 & e^{i\theta}. \end{pmatrix} \tag{60}$$

Here, n is an integer, a_m is the length of the strip unit cell, $k_l(\Omega)$ is the quasi-momentum at frequency Ω in the positive branch, $k_l(\Omega) > 0$, for an infinite strip. We note that the dispersion relation $k_l(\Omega)$ depends on the domain wall orientation at s, horizontal ($l = \mathrm{ho}$) or slanted ($l = \mathrm{sl}$). The two slanted directions are equivalent because of the mirror symmetry about the yz plane. We note that $j_{\circlearrowright}$ describes a wave propagating anticlockwise because $dk_l(\Omega)/d\Omega < 0$ in the positive quasi-momentum branch, cf. Main Text Fig. 1.

Next, we denote as s_i the coordinate of a corner. The domain wall has different orientations l and r for $s = s_i + 0_-$ (immediately before the corner) and $s = s_i + 0_+$ (immediately after the corner), respectively. We connect the solutions at the two opposite sides of the corner using the appropriate transfer matrix,

$$\begin{pmatrix} j_{\circlearrowright}(s_i+0_+) \\ j_{\circlearrowleft}(s_i+0_+) \end{pmatrix} = T^{(\mathrm{corner})}_{r,l} \begin{pmatrix} j_{\circlearrowright}(s_i+0_-) \\ j_{\circlearrowleft}(s_i+0_-) \end{pmatrix}, \quad T^{(\mathrm{corner})}_{r,l}(r,t) = \frac{1}{t_{rl}} \begin{pmatrix} \frac{t_{rl}}{t^*_{rl}} & -r^*_{rl}\frac{t_{rl}}{t^*_{rl}} \\ -r_{rl} & 1 \end{pmatrix}. \tag{61}$$

Here, r_{rl} and t_{rl} are the complex reflection and transmission coefficients, respectively, and, thus, $|r_{lr}|^2 + |t_{lr}|^2 = 1$. The form of the T-matrix above reflects the symmetry under time-reversal and the conservation of the energy flux. By applying the mirror symmetry about the yz plane to Eq. (61), one finds

$$T^{(\mathrm{corner})}_{l,r} = \frac{1}{t_{rl}} \begin{pmatrix} \frac{t_{rl}}{t^*_{rl}} & r_{rl} \\ r^*_{rl}\frac{t_{rl}}{t^*_{rl}} & 1 \end{pmatrix}. \tag{62}$$

For the special case $r = l = \mathrm{sl}$, combining the above equation with Eq. (61) gives the constraint $r^*_{\mathrm{sl\,sl}} t_{\mathrm{sl\,sl}} + t^*_{\mathrm{sl\,sl}} r_{\mathrm{sl\,sl}} = 0$. Thus, we are left with five independent transfer matrix parameters: $t_{\mathrm{ho,sl}}$, $r_{\mathrm{ho,sl}}$ (two independent phases and one independent amplitude) and $t_{\mathrm{sl,sl}}$ (independent amplitude and phase.)

Using the building blocks Eqs. (60,61) and the symmetry constraint Eq. (62) we can build a transfer matrix T_{loop} that evolves the current amplitude from a point s on a closed loop around the whole domain wall length. For example, for the triangle of side L we find the loop T-matrix

$$T_{\mathrm{loop}} = T^{(\mathrm{corner})}_{\mathrm{ho,sl}} T^{(\mathrm{edge})}_{Lk_{\mathrm{sl}}} T^{(\mathrm{corner})}_{\mathrm{sl,sl}} T^{(\mathrm{edge})}_{Lk_{\mathrm{sl}}} T^{(\mathrm{corner})}_{\mathrm{sl,ho}} T^{(\mathrm{edge})}_{Lk_{\mathrm{ho}}} \tag{63}$$

The loop T-matrix allows to impose the appropriate periodic boundary conditions, requiring

$$\det\left(T_{\mathrm{loop}} - \mathbb{1}\right) = 0. \tag{64}$$

This equations can then be solved to give the spectrum Ω_n in terms of the transfer matrix parameters. Vice versa, when the spectrum is known, one can use the same equation to extract information about the transfer matrix parameters.

Analytical calculation of the spectrum Ω_j in the absence of backscattering

We note that in the limit of zero backscattering (corresponding to the special case $r_{\rm sl,sl} = r_{\rm ho,sl} = 0$) the matrix $T_{\rm loop}$ is diagonal and as a consequence the spectrum is formed by degenerate doublets that solve the simple equation

$$L_{\rm sl} k_{\rm sl}(\Omega) + L_{\rm ho} k_{\rm ho}(\Omega) + N_{\rm sl,sl} \arg(t_{\rm sl,sl}) + N_{\rm sl,ho} \arg(t_{\rm sl,ho}) = n2\pi. \tag{65}$$

Here, $L_{\rm sl}$ ($L_{\rm ho}$) is the total length along slanted sides, and $N_{\rm sl,sl}$ ($N_{\rm sl,ho}$) is the number of corners connecting two slanted sides (a slanted and a horizontal side). Importantly, according to this formula (that assumes no backscattering) we expect the same spectrum for the two different tree-shaped topological cavity geometries. This is the tell-tale signature for the absence of backscattering (as a consequence of the topological nature of the transport) that we will be looking for in the experiment.

By deriving Eq. (65) with respect to the frequency we find a simple expression for the free spectral range between two doublets

$$\Omega_{j+2} - \Omega_j \approx \frac{2\pi v_{\rm sl} v_{\rm ho}}{L_{\rm sl} v_{\rm ho} + L_{\rm ho} v_{\rm sl}}. \tag{66}$$

where $v_{\rm sl} = d\Omega/dk_{\rm sl}$ ($v_{\rm ho} = d\Omega/dk_{\rm ho}$) is the group velocity on a slanted (horizontal) side. Here, we have assumed that the transmission amplitudes $t_{\rm sl,sl}$ and $t_{\rm sl,ho}$ are frequency independent. We note that the assumption of constant scattering parameters clearly breaks down at the crossover region between the topological and trivial region (about the edge of the horizontal strip bandwidth) where the transmission across the slanted-horizontal corners $t_{\rm sl,ho}$ must go to zero. This is the reason for the mismatch between the measured and theoretical noise spectra in that region. We do not seek to model the crossover region.

Calculation of the vacuum OM couplings g_{0n}

In this section we show how to estimate the vacuum optomechanical couplings $\{g_{0n}\}$ for the normal modes $\{\mathbf{Q}_n(\mathbf{r})\}$ of the topological mechanical cavity. By definition, this is the cavity shift by a displacement field with amplitude equal to the zero-point fluctuations. By requiring that the energy stored in the vibrations $\Omega_j^2 \int_V |\mathbf{Q}_n(\mathbf{r})|^2 \rho(\mathbf{r}) d^3\mathbf{r}$ is equal to the zero point energy $\hbar\Omega_j/2$ we find the normalization condition

$$\int_V |\mathbf{Q}_n(\mathbf{r})|^2 \rho(\mathbf{r}) d^3\mathbf{r} = \frac{\hbar}{2\Omega_n}. \tag{67}$$

The cavity shift g_{0j} is then given by the standard perturbative formulas $g_{0n} = g_{0n}^{\rm (PE)} + g_{0n}^{\rm (Bnd)}$ with the moving boundary contributions and photoelastic contributions

$$g_{0n}^{\rm (Bnd)} = -\frac{\omega_0}{2} \frac{\int_A (\mathbf{Q}_n(\mathbf{r}) \cdot \mathbf{n}) \left((\varepsilon(\mathbf{r}) - \varepsilon_0) |\mathbf{e}^{\parallel}(\mathbf{r})|^2 - (\varepsilon(\mathbf{r}) - \varepsilon_0)^{-1} |\mathbf{d}^{\perp}(\mathbf{r})|^2 \right) dA}{\int_V |\mathbf{e}(\mathbf{r})|^2 \varepsilon(\mathbf{r}) d^3\mathbf{r}} \tag{68}$$

$$g_{0n}^{\rm (PE)} = -\frac{\omega_0}{2} \frac{\int_V \mathbf{e}(\mathbf{r}) \cdot \delta\varepsilon(\mathbf{r}) \cdot \mathbf{e}(\mathbf{r})}{\int_V |\mathbf{e}(\mathbf{r})|^2 \varepsilon(\mathbf{r}) d^3\mathbf{r}} \tag{69}$$

where $\mathbf{e}(\mathbf{r})$ and $\mathbf{d}(\mathbf{r})$ are the electric and the electric displacement fields in the cavity mode, respectively. Moreover, $\varepsilon(\mathbf{r})$ is the permittivity, and the tensor $\delta\varepsilon(\mathbf{r})$ is the local change of permittivity due to the strain. For the purpose of our discussion it is only important that the mapping between the displacement field $\mathbf{Q}_n(\mathbf{r})$ and the tensor $\delta\varepsilon(\mathbf{r})$ (via the strain tensor) is linear, see Ref. [10] for more details. Since the electric and the displacement fields are exponentially localized within a single triangular membrane, we can approximate the displacement field as

$$\mathbf{Q}_n(\mathbf{r}) \approx A_{n,i} e^{ik_l(\Omega)s} \mathbf{u}_{n,l,\Omega_n}(\mathbf{r}) + A_{n,i}^*(s) e^{-ik_l(\Omega)s} \mathbf{u}_{n,l,\Omega_n}^*(\mathbf{r}), \tag{70}$$

where $\exp[ik_l(\Omega)s]\mathbf{u}_{n,l,\Omega_n}(\mathbf{r})$ are the edge state Bloch waves for an infinite strip with the appropriate orientation. Thus, $\mathbf{u}_{n,l,\Omega_n}(\mathbf{r})$ is a periodic function in the domain wall direction, e.g. it is periodic in x for $l = \text{ho}$, and is exponentially localized about the domain wall in the transverse direction. The complex amplitude $A_{n,i}$ is assumed to be constant

in the region of the cavity but its value, to be calculated using the scattering matrix approach and imposing the normalization condition Eq. (67), may depend on the side of the polygon-shaped domain wall labeled by the second index i, see below.

Next, we define the vacuum 1D optomechanical coupling (per unit cell) $g_{0,l}^{(1D)}(\Omega)$ (the quantity plotted in Fig. 2 of the Main Text) as the cavity shift in the presence of a 1D Bloch wave $\mathbf{u}_{n,l,\Omega}(\mathbf{r})$ of amplitude set by the normalization condition

$$\int_U |\mathbf{u}_{n,l,\Omega_n}(\mathbf{r})|^2 \rho(\mathbf{r}) d^3\mathbf{r} = \frac{\hbar}{2\Omega_n}, \tag{71}$$

where U indicates the unit cell of the strip. This definition has the merit to be independent of the size of the system (length of the strip) and is, thus, suitable to be computed using finite element simulations. We note that we can fix the phase of $\mathbf{u}_{n,l,\Omega}(\mathbf{r})$ by requiring that $g_{0,l}^{(1D)}(\Omega)$ is real. Using this definition and substituting Eq. (70) into Eq. (68), we find

$$g_{0n}(s) = g_{0,l}^{(1D)}(\Omega_n) 2|A_{n,i}| \cos(k_l(\Omega)s + \arg(A_{n,i})) \tag{72}$$

where s is the position of the cavity. Thus, the task of calculating the optomechanical coupling reduces to the task of calculating the amplitude $A_{n,i}$. This is done by noting that using Eq. (70), we can identify the amplitude of the clockwise and anti-clockwise mechanical energy fluxes with

$$j_{\circlearrowright}(s) = \left(v_l \frac{\hbar\Omega_n}{2a_m}\right)^{1/2} A_{n,i} e^{ik_l(\Omega)s}, \quad j_{\circlearrowleft}(s) = \left(v_l \frac{\hbar\Omega_n}{2a_m}\right)^{1/2} A_{n,i} e^{-ik_l(\Omega)s}, \tag{73}$$

respectively. Thus, we can calculate $A_{n,i}$ from Eq. (64) modulus a normalization factor (the global phase of the solution of Eq. (64) does not have a physical meaning and is fixed by requiring that $j_{\circlearrowright}(s) = j^*_{\circlearrowleft}(s)$. This is always possible because of the time-reversal symmetry). The normalization factor is fixed by Eq. (67). Using Eq. (70) and Eq. (71), the latter constraint can be rewritten as

$$1 = \sum_i 2|A_{n,i}|^2 N_i, \tag{74}$$

where N_i is the length of the side i (in number of unit cells).

For the case of zero backscattering the flux $|j_{\circlearrowright}(s)|$ is constant, cf. Eq. (64). Thus, from Eq. (73) and Eq. (74) we find

$$|A_{n,\mathrm{ho}}| = \left(\frac{a_m v_{\mathrm{sl}}}{2(L_{\mathrm{ho}} v_{\mathrm{sl}} + L_{\mathrm{sl}} v_{\mathrm{ho}})}\right)^{1/2}, \quad |A_{n,\mathrm{sl}}| = \left(\frac{v_{\mathrm{ho}}}{v_{\mathrm{sl}}}\right)^{1/2} |A_{n,\mathrm{ho}}|. \tag{75}$$

where $|A_{n,\mathrm{ho}}|$ ($|A_{n,\mathrm{sl}}|$) is the amplitude on all horizontal (slanted) sides. We note that for a pair of degenerate solutions have the same amplitudes, $|A_{n,\mathrm{l}}| = |A_{n+1,\mathrm{l}}|$, while $|\arg(A_{n,\mathrm{l}}/A_{n+1,\mathrm{l}})| = \pi/2$ to ensure that the two solutions are orthogonal.

It is interesting to estimate the typical amplitude of the zero-point and the thermal fluctuations in our topological cavity. For the zero point fluctuations we have [10]

$$x_{\mathrm{zpf,n}} = \mathrm{Max}[\mathbf{Q}_n(\mathbf{r})]_V \approx |A_{n,\mathrm{ho}}| \mathrm{Max}[\mathbf{u}_{n,l,\Omega_n}(\mathbf{r})]_U \approx N^{-1/2} \mathrm{Max}[\mathbf{u}_{n,l,\Omega_n}(\mathbf{r})]_U \tag{76}$$

We calculate the quantity $\mathrm{Max}[\mathbf{u}_{n,l,\Omega_n}(\mathbf{r})]_U$ using FEM simulations. It turns out that $\mathrm{Max}[\mathbf{u}_{n,l,\Omega_n}(\mathbf{r})]_U \sim 1\mathrm{fm}$. Taking into account that for our devices $N \sim 100$ we find

$$x_{\mathrm{zpf,n}} \sim 0.1\mathrm{fm}. \tag{77}$$

We can then readily find the typical amplitude of the thermal vibrations in our experiment to be

$$x_{\mathrm{th}} \sim \sqrt{\frac{k_B T}{\hbar\bar{\Omega}}} x_{\mathrm{zpf,n}} \sim 10\mathrm{fm}. \tag{78}$$

Moreover, we can estimate the displacement sensitivity of our measurement setup from the measured NPSD of the mechanical waves, see Supplementary Figure 4. The sensitivity of our setup is limited by the background NPSD $S_{\mathrm{mech,bg}}$ (this is the sum of the photon shot noise and any technical noise, referred back to the input). The sensitivity

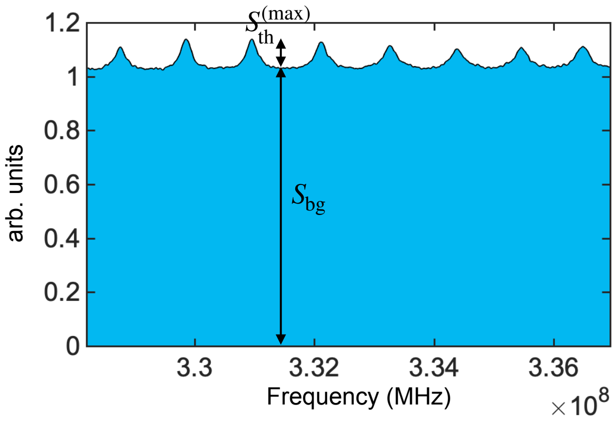

Supplementary Figure 4. **Mechanical noise spectrum.** Measured NPSD as a function of frequency (inside the bulk band gap, in the topological region) on the slanted edge of the triangular mechanical cavity.

can be evaluated from the measurement signal in Supplementary Figure 4, using for calibration the mechanical thermal noise $S^{(\rm max)}_{\rm mech,th} = 8x^2_{\rm th}/\Gamma$ (see Eq. (58)). For this purpose, we take advantage of the fact that any (potentially unknown) calibration scale factors drop out when taking the ratio between the background noise level and the amplitude of the thermal peak on top of it. Thus, we can set equal this ratio, as obtained from theory (left-hand-side of the following equation), to the same ratio as deduced from the experimental numbers:

$$\frac{S_{\rm mech,bg}}{S^{(\rm max)}_{\rm mech,th}} = \frac{S_{\rm bg}}{S^{(\rm max)}_{\rm th}}. \tag{79}$$

Note that the ratio on the right-hand-side, $S_{\rm bg}/S^{(\rm max)}_{\rm th}$, can be directly read out from the measurement signal. In this way, we obtain the sensitivity as $\sqrt{S_{\rm mech,bg}} \approx 8 \times 10^{-17}\,\rm m/\sqrt{Hz}$ ($\sqrt{\Gamma S_{\rm mech,bg}} \approx 90\,\rm fm$). Thus, in a single-shot measurement (not actually implemented in the present work), we would be able to pinpoint the position quadrature amplitude of the mechanical oscillator up to a precision of 90 fm in a measurement time Γ^{-1} (corresponding to the typical decay time of the vibrations).

Note that in the present experiment we are interested not in performing single-shot measurements, but only in measuring thermal noise spectra, which involves averaging over arbitrarily long times. This allows us to measure the spectra of thermal vibrations with amplitudes on the order of 10fm.

Fitting parameters

In calculating the spectrum Ω_n and the corresponding optomechanical couplings g_{0n}, we have made the simplifying assumption that the propagation along the domain wall is similar as the propagation in an infinitely long domain wall and abruptly switch to the propagation in a domain wall with a different orientation after turning a sharp angle. This allows for a simple theoretical description but is not entirely realistic in the region close to the corners. A more realistic point of view is that Eq. (60) is valid only away from the corners and Eq.(61) describes the propagation across a finite region about the corners. In view of this physical interpretation, we replace in our calculation of the noise spectrum the lengths of the sides of the polygon-shaped domain wall with effective lengths that are determined using the total length of the domain wall as a fitting parameter and rescaling accordingly the single side lengths.

For the plots where we have assumed perfect transmission we take also the overall phase acquired by crossing the three corners (for the triangle $\arg(t_{\rm sl,sl} + 2t_{\rm sl,ho})$) as a fitting parameter.

In addition we have used a uniform shift of the band structure as a fitting parameter. This is justified because, within the topological bandwidth of our devices, the main effect of a rescaling of the whole band structure by as little as $\sim 1\%$ would be a uniform shift of the order of the free spectral range. This could be caused by any residual mismatch between the fabrication and the nominal parameters used for our FEM simulations. Indeed, we have observed a uniform shift of the band structure even post fabrication comparing measurement taken on the same device in different days. We attribute this drift, that tends to saturate after some time, to oxidation of the device surface.

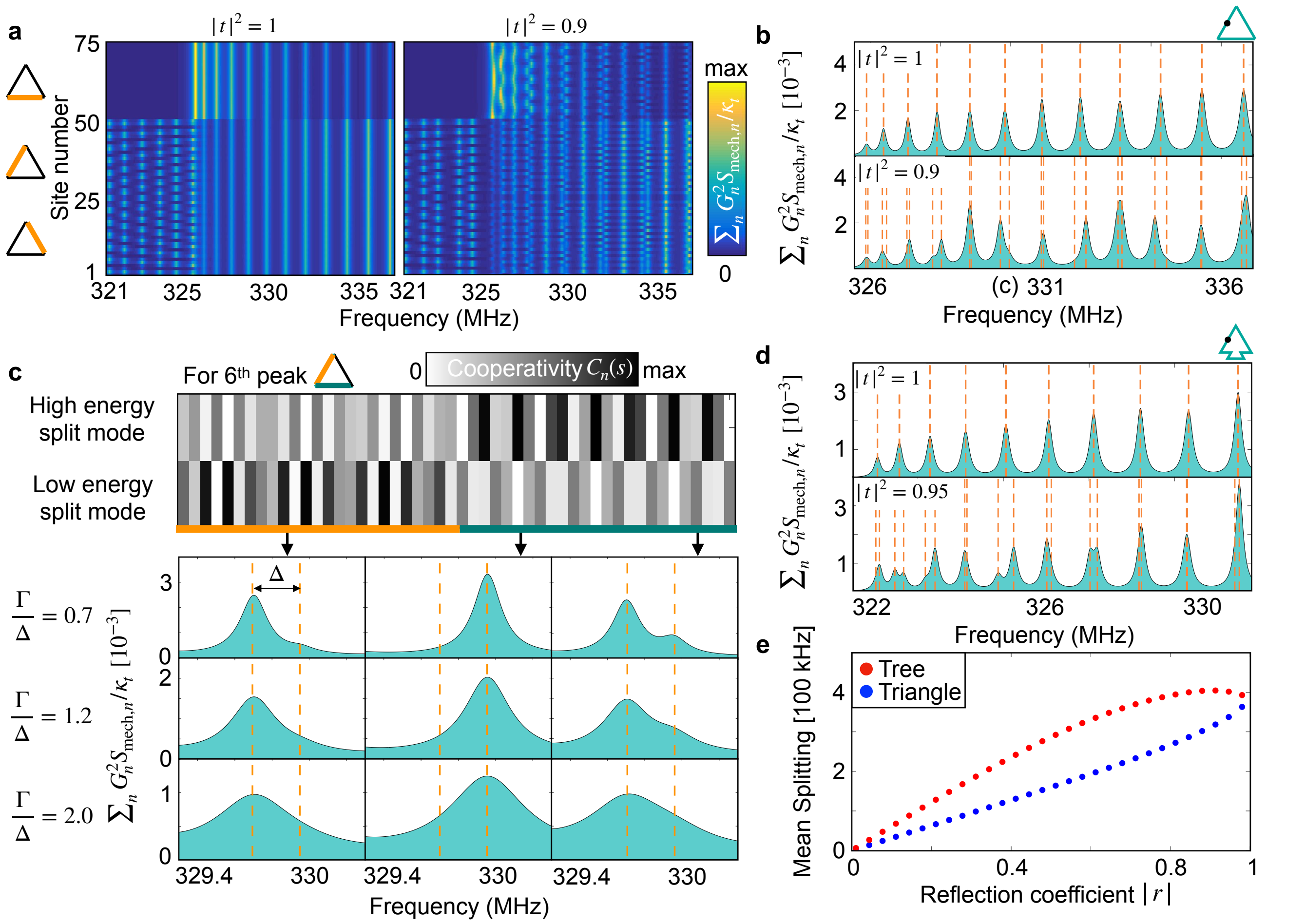

Supplementary Figure 5. **Signatures of backscattering on the spectrum. a**, Theoretically predicted spectra on all the sites of the triangular topological mechanical cavity (shown in the form of a density plot). For non-vanishing backscattering $|t|^2 = 0.9$, the spectra become position-dependent, i.e. they are no longer identical for all the sites on the slanted or the horizontal waveguide. **b** (**d**), Local spectrum for triangular (tree) topological mechanical cavity at the middle of the long slanted waveguide, in the absence and presence of small backscattering. The orange dashed lines indicate the eigenvalues. In the presence of small backscattering, the degenerate peaks are split and the amplitudes of the peaks are modulated. **c**, Intensity mode profile (displayed in terms of optomechanical cooperativity) of the two split modes of the 6th doublet peak for the triangular topological mechanical cavity. Resulting spectrum around this peak at different sites for small, intermediate and large values of mechanical damping Γ. **e**, Comparison of the mean splitting for the triangular and tree topological mechanical cavities. The splitting is larger for the tree cavity because it has more corners than the triangular cavity.

Effect of finite backscattering on the spectrum

Here, we study the effect of backscattering on the NPSD. As discussed above in order to calculate the NSPD we first have to calculate the eigenfrequencies Ω_n and the optomechanical couplings g_{0n} in the transfer matrix approach using Eqs. (64,74,72). Then, we can plug these quantities into the general expression Eq. 57. For simplicity we always assume $t_{\text{sl,sl}} = t_{\text{sl,ho}} = t$.

Supplementary Figure 5(a) shows the triangle NPSD for $|t|^2 = 0.9$ (right). For comparison, we also show the results calculated assuming perfect transmission, $|t|^2 = 1$ (left).

In the presence of backscattering $\left(|r|^2 \neq 0\right)$, the underlying doublets Ω_n and Ω_{n+1} (indicated by orange lines in Supplementary Figure 5 (b) and (d)) are split. The splitting averaged over all doublets as a function of $|r|$ is plot in Supplementary Figure 5 (e). We note that the average splitting is always larger for the tree-shaped topological cavity than for the triangular cavity. This should be expected because of the larger number of corners acting as scattering centers for the tree geometry. The splitting of the underlying doublets changes the lineshape of the NPSD. The position dependence of the lineshape is governed by the ratio of the splitting and the decay rate and is studied

for the 6-th peak in Supplementary Figure 5 (c). We note that for splittings Δ similar to the decay rate Γ (top and center panel), we see either one or two peaks in the spectrum, depending on the position dependence of the intensity profiles of the two modes (via the position dependent optomechanical couplings, cf. Eq. (72)) at the measurement location. On the other hand, in the regime of our experiment where the splitting Δ is smaller than the decay rate Γ, there is always a single peak. In this regime the residual small splitting Δ is revealed by what looks like a position dependent drift of the peak location.

Supplementary Note 8. CALIBRATION OF FREQUENCY DRIFT AND NORMALIZATION OF NPSD SPECTRA OF THE MECHANICAL TOPOLOGICAL CAVITY

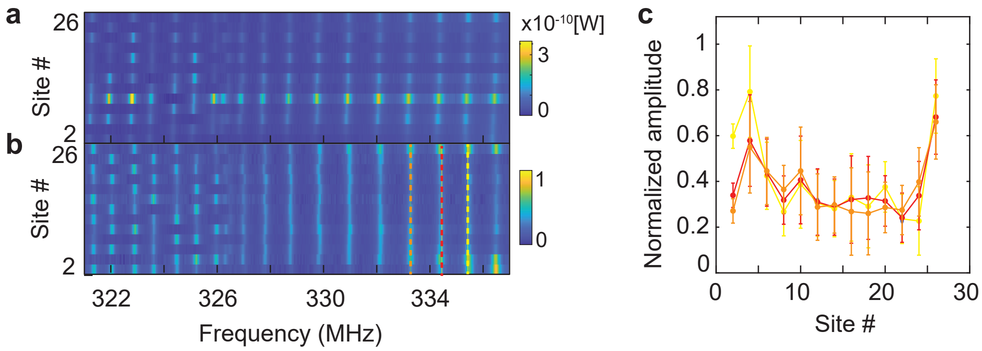

Supplementary Figure 6. **Calibration of frequency drift and normalization of NPSD spectra of the mechanical topological cavity a**, Density plot of the measured NPSD before frequency drift calibration amplitude normalization, as a function of frequency and read-out position along a slanted edge. **b**, Density plot of the same measured NPSD after frequency drift calibration and amplitude normalization. **c**, Normalized peak amplitudes verses measurement locations for 3 peaks, corresponding frequencies are indicated with dashed lines in **b**. Error bars correspond to the standard deviation of the 5 peak amplitudes used for the amplitudes normalization.

We have observed a uniform shift of the band structure during measurements comparing spectra taken on the same device on different days. We attribute this drift, that tends to saturate after some time, to surface oxidation of the silicon device. In addition, the total optical loss κ, external coupling efficiency κ_e/κ, as well as the total optical optical power delivered to each optical cavity during measurements are different, hence the amplitude of the optically transduced mechanical NPSD received on spectrum analyzer are different for each site. Therefore, we have applied spectra normalization, as discussed below, for the Main Text figures Fig.2 and Fig.3.

The frequency drift is calibrated using the two peaks between 323 MHz and 325 MHz. The frequency spectra of all the sites are shifted such that these two peaks align as well as possible with the corresponding two peaks at the (arbitrarily selected) reference site #26. To illustrate this procedure, the raw spectra are plotted in Supplementary Figure 6a; it can be seen that there is a drift in mechanical frequency to lower frequencies from site #26 to site #2. In Supplementary Figure 6b, the frequency drifts have been removed by the calibration.

The amplitudes of the measured spectra are also normalized. Essentially, there are differences in the intensities between measurements at different sites due to these sites being measured individually in succession, with different κ, κ_e/κ, and total optical optical power delivered to the optical cavity during measurements. These spurious differences (that are not connected to the intrinsic physics of the device) are removed by calibration in the following manner. The five highest peaks in the grey low-frequency region (cf. Main Text Fig. 1i) are averaged, and the mean value is used to normalize each spectrum. The theoretical expectations for the NPSD as a function of frequency and read-out position on a slanted edge are plotted in Supplementary Figure 5a. Figure 6 shows the normalized peak amplitudes versus measurement location for 3 peaks. It can be seen that the normalized NPSD near the center of the slanted side of the triangle mechanical topological cavity are constant within error.

We remark briefly on why we have chosen our calibration procedure in this way. Our aim in this site-resolved measurement was to test experimentally whether the high-frequency region indeed harbors running wave modes that have little to no backscattering, as expected from the topological nature of the edge channels. Such modes would then show up with an intensity that is independent of position (in the idealized absence of changes of coupling strength between the measurements at different sites). In order to test this hypothesis without bias, we decided against normalizing the overall intensity based on peaks in this high-frequency region (because this could be viewed

as enforcing at least an average tendency towards location-independent intensities). We rather took peaks in the low-frequency region as a reference for the normalization. Since in that region we expect standing waves (due to backscattering at the ends of the edge), each individual frequency peak is already strongly location-dependent in its intensity. To avoid hampering the overall normalization by this fact, we averaged over the five highest peaks.

Supplementary Note 9. CALIBRATION OF THE OPTOMECHANICAL COUPLING RATE

The optomechanical coupling of the between localized optical resonance and mechanical resonance can be estimated using two different methods. The first method, the increase in the mechanical linewidth of each mechanical mode can be fitted as a function of optical power to find the optomechanical coupling for each mode. However, the increased mechanical linewidth of our devices is on the order of few hundred Hz ($\gamma_{\mathrm{OM}} < 1$ kHz), even with large number of cavity photons ($n_{\mathrm{c}} \approx 10000$), which is small compared to the intrinsic mechanical linewidth ($\gamma_{\mathrm{i}} \approx 200$ kHz). The second method involves calibration of the optical powers and electronic detection system, and uses the fact that the transduced thermal Brownian motion of the mechanical resonator is proportional to $g_{0\mathrm{n}}^2$.

To calibrate the detection efficiency of the setup we first measure the efficiency of transmission from laser to the input power of the dimpled fiber taper. These values are measured once when the optical components are connected and do not change. To measure the efficiency of dimpled fiber taper, the laser is tuned off-resonance from the optical mode (where the device optical transmission should be flat) and a continuous-wave signal of input power P_{in} is sent into input port of the dimpled fiber taper. The optical losses incurred in the path input port of taper to the device-under-test are accumulated into an efficiency factor η_{taper}. These losses are incurred twice in transmission to the output of the taper (loss in the input side of taper is assumed to be the same as output side), so a power of $\eta_{\mathrm{taper}}^2 P_{\mathrm{in}}$ propagates out of output port of the taper. This signal is sent to a power meter (PM), and thus the coupling efficiency of taper is determined as $\eta_{\mathrm{taper}} = \sqrt{P_{\mathrm{PM}}/P_{\mathrm{in}}} = 51\%$.

To calibrate the overall detection efficiency (η_{det}), we must also determine the efficiency of the rest of the detection path and detector. This is accomplished by using the amplitude modulator to create optical sidebands detuned from the signal by the mechanical frequency while the laser is tuned off-resonance from the optical mode. The power P_{cal} in this sideband is calculated using V_π of EOM and η_{taper}. The photocurrent NPSD ($S_{\mathrm{pc}}[\omega]$) as transduced on the spectrum analyser is given by

$$S_{\mathrm{pc}}[\omega] = S_{\mathrm{dark}} + \frac{G_{\mathrm{e}}^2}{R_{\mathrm{L}}} S_{\mathrm{SN}}^2 \left(1 + \frac{\eta_{\mathrm{det}} S_{\mathrm{cal}}[\omega]}{\hbar\omega_{\mathrm{o}}}\right), \tag{80}$$

where $S_{\mathrm{dark}}[\omega]$ is the electronic NPSD of the detector, $S_{\mathrm{SN}} = \sqrt{2\hbar\omega_{\mathrm{o}} P_{\mathrm{drive}}}$ is the optical shot-noise NPSD arising from driving optical power at optical frequency ω_{l}, which lies an order of magnitude above the electronic noise, and S_{cal} is the NPSD of the signal, where $\int_{-\infty}^{\infty} S_{\mathrm{cal}}[\omega]\frac{d\omega}{2\pi} = \eta_{\mathrm{taper}}^2 P_{\mathrm{cal}}$. The gain factor G_{e} represents the conversion from optical power to voltage while R_{L} is the input impedance of the spectrum analyser. The total noise floor $S_{\mathrm{noise}} = \frac{G_{\mathrm{e}}^2}{R_{\mathrm{L}}} S_{\mathrm{SN}}^2 + S_{\mathrm{dark}}$ is measured with the EOM drive turned off (no optical sidebands), while S_{dark} is measured independently with both signal and drive laser beams blocked (laser is blocked at BOA). The calibration tone (with NPSD $S_{\mathrm{cal}}[\omega]$) picks up losses in the optical setup (fibers, fiber unions), fast optical detector, and microwave cable, which are parametrized into η_{det}. The efficiency of the thermal vibration NPSD detection path is extracted as

$$\eta_{\mathrm{det}} = \frac{\hbar\omega_{\mathrm{o}}}{\eta_{\mathrm{taper}}^2 P_{\mathrm{cal}}} \int_{-\infty}^{\infty} \frac{S_{\mathrm{pc}}[\omega] - S_{\mathrm{noise}}}{S_{\mathrm{noise}} - S_{\mathrm{dark}}} \frac{d\omega}{2\pi} = 9.7\%. \tag{81}$$

In order to calibrate $g_{0\mathrm{n}}$, the fiber taper is parked on the device-under-test, which is one of the center sites of slanted edge of a tree-shaped cavity geometry in this calibration. The optical drive laser is locked to a blue detuning (from the optical cavity resonance) of 340 MHz, and the optical drive power is tuned such that the same amount of power is received on the photodetector. With

$$S_{\mathrm{pc}}[\omega] = S_{\mathrm{dark}} + \frac{G_{\mathrm{e}}^2}{R_{\mathrm{L}}} S_{\mathrm{SN}}^2 \left(1 + \frac{\eta_{\mathrm{det}} S_{\mathrm{II}}[\omega]}{\hbar\omega_{\mathrm{o}}}\right), \tag{82}$$

where S_{II} is the NPSD of optomechanically generated photons, given by Eq. 57. For the tree-shaped topological cavity, we get calibrated $g_{0\mathrm{n}}/2\pi = 2.56$ kHz for one peak (328.21 MHz) within topological bandwidth, which is in good agreement with the theoretically predicted value (Eq. 72) for the same peak, $g_{0\mathrm{n}}/2\pi = 2.25$ kHz. We note that this agreement implies that we are indeed observing thermal motion (S_{II} in Eq. 57 is calculated for the room temperature 300 K).

Supplementary Note 10. TRIVIAL WAVEGUIDE DESIGN

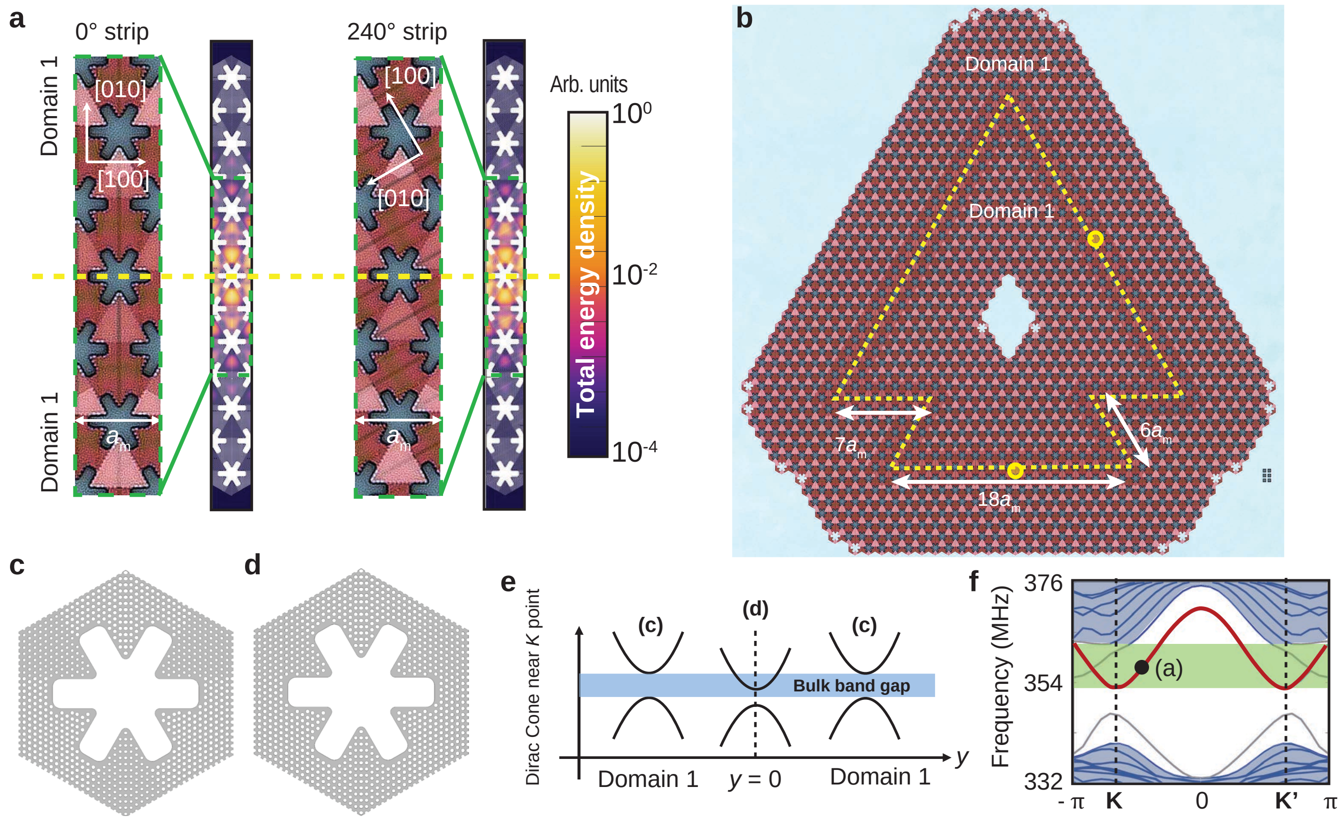

Supplementary Figure 7. **Trivial waveguide a**, Optical microscope images and simulated mechanical mode profiles of the strip unit cell for 0° (horizontal) or 240° (slanted) vs. the silicon [100] crystal orientation. A defect trivial waveguide is built by modifying the radius of the holes in the upward-pointing triangles near the center three snowflakes. **b**, Optical microscope image of the tree-shaped trivial mechanical cavity. The dimensions are similar to that of topological mechanical cavity in Fig. 3(b) of Main Text. The center of trivial waveguide is indicated by dashed lines. Mechanical NPSD is measured at the horizontal and slanted edges of tree geometry as indicated by the yellow dots. **c**, Unit cell in domain 1. **d**, Unit cell of the center three snowflakes. **e**, The Dirac cone bands of the center three snowflakes are tuned into the bulk band gap by changing the size of the circular holes in the upward-pointing triangles. Here we display the Dirac cones of the waveguide region in the center plot, and the two outer plots show the Dirac cones in the surrounding bulk crystal. The blue shaded region is the gap between the Dirac cones in the surrounding bulk region of the phononic crysta.l **f**, 1-D band structures calculated for the horizontal configuration of the trivial waveguide. The green shaded region is the frequency gap between the Dirac cones in the (unperturbed) surrounding bulk phononic crystal. The red curve is the dispersion relation of the guided mode pulled into the bulk gap; the grey lines are the additional edge state modes localized at the top and bottom boundaries of the geometry (away from the domain wall); the blue shaded regions are bulk modes.

In this section, we describe the design of the trivial waveguide, which is used in Fig. 3 of the Main Text to show the effects of backscattering at the sharp corners. A trivial waveguide is formed by locally tuning the upper Dirac cone band to be inside that of the bulk Dirac cone gap of the surrounding bulk phononic crystal. This local trivial waveguide region consists of a row of three snowflakes unit cells; the corresponding optical microscope images and simulated mechanical mode profiles of 0° and 240° of the strip unit cells vs. Silicon [100] are shown in Supplementary Figure 7(a). In the center three snowflake unit cells, the circular holes in the upward-pointing triangles (light red) are scaled smaller by a factor of 0.92 in comparison to the downward-pointing triangles (See Supplementary Figure 7(d)). This should be compared to the surrounding bulk region (domain 1), where circular holes in the upward-pointing triangles are scaled smaller by a factor of 0.78 (see Supplementary Figure 7(c)). Larger circular holes shift the gapped Dirac cones lower in energy because of the decreasing stiffness (see Supplementary Figure 7(e)). Modes in the top Dirac cone are thus pulled into the Dirac cone gap of the bulk region (domain 1), forming a localized waveguide mode as desired. The dispersion of the trivial waveguide mode is shown as a red curve in Supplementary Figure 7(e) for a

horizontal section of the waveguide. Note that this dispersion curve is fundamentally different than the topological edge mode dispersion shown in Fig. 1(e) of the Main Text, in which at the **K** and **K′** there is only one edge mode state with a single group velocity direction (the two edge modes have opposite group velocity). In the trivial guided mode dispersion of Supplementary Figure 7(e), both **K** and **K′** points correspond to a dispersion minimum. There are thus two guided modes with opposite group velocity in the **K** and **K′** valley regions for the trivial waveguide. Scattering between forward and backward guided modes can thus occur with very small quasi-momentum transfer within a given valley. This is the fundamental reason that the topological edge modes are protected against backscattering; there is no forward and backward mode within the same valley.

An optical microscope image of the tree cavity geometry is shown in Supplementary Figure 7(b). In the data presented in Fig. 3 of the Main Text, the modes of the tree-shaped trivial mechanical cavity are optically readout at the horizontal and slanted edges of tree geometry as indicated by yellow dots.

Supplementary Note 11. EFFECT OF DISORDER ON THE BACKSCATTERING OF THE EDGE STATE

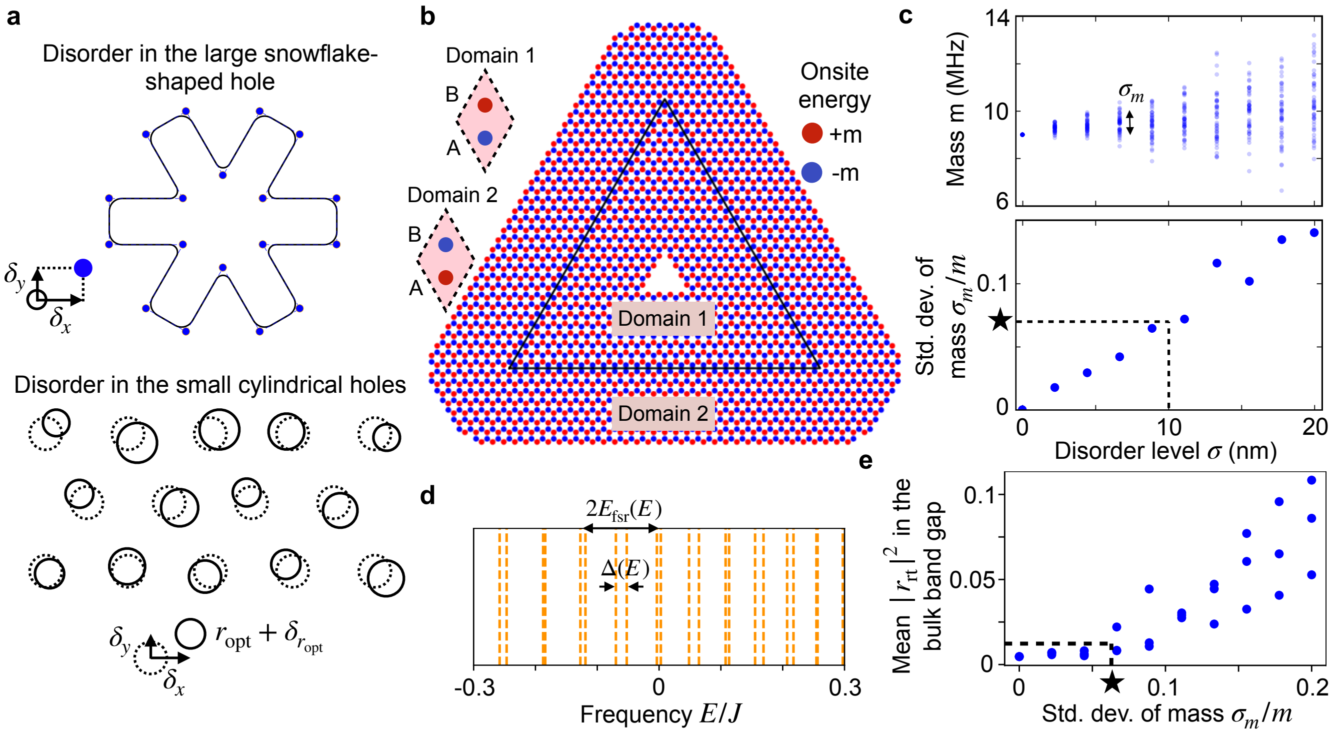

Supplementary Figure 8. **Effect of disorder on the backscattering of the edge state a**, Geometrical disorder considered for the FEM simulation of the bulk unit cell. For the large snowflake holes, each vertex (blue) is randomly displaced about the ideal location by δ_x and δ_y in the two directions. Moreover, for the small cylindrical holes, we consider disorder in both their central locations and the radii. The disorder variables are assumed to be independent and identically sampled from a Gaussian distribution with zero mean and standard deviation σ. **b**, Tight-binding model of the triangular topological cavity. The onsite energies on the two sublattices A and B are exchanged for the two domains (inset). **c**, (top) Relation between the disorder level σ and the mass parameter (half of the bulk band gap) obtained via FEM simulations of the different disorder realizations of the bulk unit cell. (bottom) Normalized standard deviation of mass σ_m/m as a function of the disorder level σ. The 'star' indicates σ_m/m for the disorder level of $\sigma = 10$ nm. **d**, Eigenvalues of the tight-binding Hamiltonian of the mechanical cavity near the center of the bulk band gap (analogous to the Supplementary Figures 5(b) and (d)) for $\sigma_m/m = 0.2$. **e**, Mean of the round trip reflection probability $|r_{\rm rt}(E)|^2$ over all the mode-splittings near the center of the bulk band gap $E/J \in [-0.3, 0.3]$ as a function of σ_m/m. The three dots at each σ_m/m correspond to the different realizations of the disorder in the tight-binding model.

In this section, we explore the effect of the fabrication-induced disorder on the backscattering of the topologically protected edge states in the triangular mechanical cavity (Fig. 2 of the Main Text). In the Valley Hall effect, the two counter-propagating edge states are located at the two valleys in the quasi-momentum space. Therefore, the

backscattering can only occur if the disorder strength is sufficiently strong to impart a large quasi-momentum transfer between the two valleys.

In our setup, the fabrication-induced disorder can either be systematic or random. Systematic disorder involves a gradient of the difference between the fabricated and ideal geometry along the array, whereas random disorder involves the random fluctuation of the fabricated geometry about the ideal one. We do not observe any clear signatures of systematic disorder in the experiment. Therefore, in this section, we study qualitatively the effect of random disorder on the transport of the edge states. During fabrication, random disorder can occur due to the geometrical fluctuations of the large snowflake holes and the small cylindrical holes, see Supplementary Figure 8(a). An ideal way to estimate the effect of the disorder on the backscattering would be to perform a full-scale FEM simulation of the triangular mechanical cavity for the different random geometry realizations. However, it is impracticable to implement such a large simulation, therefore we devise an indirect approach of estimating the backscattering in the simpler case of a tight-binding model (whose parameter fluctuations we connect to the microscopic disorder-induced fluctuations).

The Valley Hall effect is usually represented on the graphene tight-binding model with different onsite energies on the two sublattices, labeled as A and B. In this setting, the two different topological configurations (domains) are obtained by exchanging onsite energies on the two sublattices, cf. Supplementary Figure 8(b). This transformation corresponds to a mirror symmetry operation about the horizontal axis. The tight-binding Hamiltonian of the topological triangular cavity is given by

$$\hat{H} = \sum_{\mathbf{x}} (m(\mathbf{x}) + \delta_{m,a}(\mathbf{x}))\, \hat{a}^{\dagger}_{\mathbf{x}} \hat{a}_{\mathbf{x}} - (m(\mathbf{x}) + \delta_{m,b}(\mathbf{x}))\, \hat{b}^{\dagger}_{\mathbf{x}} \hat{b}_{\mathbf{x}} - J \sum_{\langle \mathbf{x}, \mathbf{x}' \rangle} \hat{a}^{\dagger}_{\mathbf{x}} \hat{b}_{\mathbf{x}'} + \hat{b}^{\dagger}_{\mathbf{x}} \hat{a}_{\mathbf{x}'}. \tag{83}$$

Here, the operators $\hat{a}$ ($\hat{a}^{\dagger}$) and $\hat{b}$ ($\hat{b}^{\dagger}$) annihilate (create) a phonon on the sublattices A and B respectively, $\mathbf{x}$ is the position vector of the unit cell, the symbol $\langle \mathbf{x}, \mathbf{x}' \rangle$ indicates the nearest-neighbor interaction, and $m(\mathbf{x}) = +m$ for $\mathbf{x} \in$ domain 2 ($-m$ for domain 1). For the tight-binding model, the 1-D strip bandstructure features an edge state with velocity $v = \sqrt{3}/2Ja_m$ (a_m is the lattice vector) in the bulk band gap of $\Delta E = 2m$. Using this information, we can fit the mass parameter for our experiment to be $m = 0.58J$. The disorder in the onsite potentials of the two sublattices is simulated by the random variables $\delta_{m,a}$ and $\delta_{m,b}$ respectively, which are sampled from a Gaussian distribution with mean zero and standard deviation σ_m. Note that we assume that the random variables between different sites are uncorrelated. The relation between σ_m and the random geometrical disorder can be estimated from the FEM simulation of the bulk geometry, see Supplementary Figure 8(c). The eigenstates of the above Hamiltonian near zero frequency represent the domain wall modes of the mechanical cavity, see Supplementary Figure 8(d). The round trip reflection probability $|r_{\text{rt}}|^2$ of the wave packet at energy E can be roughly estimated from the mode-splitting $\Delta(E)$ and the free spectral range $E_{\text{fsr}}(E)$ using the relation $|r(E)|^2 \approx |\Delta(E)/E_{\text{fsr}}(E)|^2$. Thus, for the standard deviation in the fabrication disorder of 10 nm, we estimate a round trip reflection probability $|r_{\text{rt}}|^2$ for the triangular mechanical cavity of around 1.25%, see Supplementary Figure 8(d,e). We expect the fabrication disorder using our electron-beam lithography to be in the range of $2 - 4$ nm [11].

SUPPLEMENTARY REFERENCES

[1] COMSOL Multiphysics 5.3a, http://www.comsol.com/.

[2] Sekoguchi, H., Takahashi, Y., Asano, T. & Noda, S. Photonic crystal nanocavity with a q-factor of 9 million. *Opt. Express* **22**, 916–924 (2014).

[3] Johnson, S. G., Villeneuve, P. R., Fan, S. & Joannopoulos, J. D. Linear waveguides in photonic-crystal slabs. *Physical Review B* **62**, 8212 (2000).

[4] Chutinan, A. & Noda, S. Waveguides and waveguide bends in two-dimensional photonic crystal slabs. *Physical review B* **62**, 4488 (2000).

[5] Huang, H., Zhou, S. & Duan, W. Type-ii dirac fermions in the $\mathbf{ptse_2}$ class of transition metal dichalcogenides. *Phys. Rev. B* **94**, 121117 (2016).

[6] Asbóth, J. K., Oroszlány, L. & Pályi, A. *A Short Course on Topological Insulators: Band Structure and Edge States in One and Two Dimensions*. Lecture Notes in Physics (Springer International Publishing, 2016).

[7] Brendel, C., Peano, V., Painter, O. J. & Marquardt, F. Pseudomagnetic fields for sound at the nanoscale. *Proceedings of the National Academy of Sciences* **114**, E3390–E3395 (2017).

[8] Hopcroft, M. A., Nix, W. D. & Kenny, T. W. What is the young's modulus of silicon? *Journal of Microelectromechanical Systems* **19**, 229–238 (2010).

[9] Aspelmeyer, M., Kippenberg, T. J. & Marquardt, F. Cavity optomechanics. *Rev. Mod. Phys.* **86**, 1391–1452 (2014).

[10] Safavi-Naeini, A. H. & Painter, O. Optomechanical crystal devices. In Aspelmeyer, M., Kippenberg, T. J. & Marquardt, F. (eds.) *Cavity Optomechanics*, Quantum Science and Technology, 195–231 (Springer Berlin Heidelberg, 2014).

[11] MacCabe, G. S. *et al.* Nano-acoustic resonator with ultralong phonon lifetime. *Science* **370**, 840–843 (2020).
